# Slow integrin-dependent migration organizes networks of tissue-resident mast cells

**DOI:** 10.1038/s41590-023-01493-2

**Published:** 2023-04-20

**Authors:** Lukas Kaltenbach, Paloma Martzloff, Sarah K. Bambach, Nadim Aizarani, Michael Mihlan, Alina Gavrilov, Katharina M. Glaser, Manuel Stecher, Roland Thünauer, Aude Thiriot, Klaus Heger, Katrin Kierdorf, Stephan Wienert, Ulrich H. von Andrian, Marc Schmidt-Supprian, Claus Nerlov, Frederick Klauschen, Axel Roers, Marc Bajénoff, Dominic Grün, Tim Lämmermann

**Affiliations:** 1 Max Planck Institute of Immunobiology and Epigenetics, Freiburg, Germany; 2 International Max Planck Research School for Immunobiology, Epigenetics and Metabolism (IMPRS-IEM), Freiburg, Germany; 3 Faculty of Biology, University of Freiburg, Freiburg, Germany; 4 Advanced Light and Fluorescence Microscopy Facility, Centre for Structural Systems Biology (CSSB) and University of Hamburg, Hamburg, Germany; 5 Leibniz Institute of Virology (LIV), Hamburg, Germany; 6 Department of Immunology and HMS Center for Immune Imaging, Harvard Medical School, Boston, MA USA; 7 The Ragon Institute of MGH, MIT and Harvard, Cambridge, MA USA; 8 Department of Cancer Immunology, Genentech, South San Francisco, CA USA; 9 Institute of Neuropathology, Faculty of Medicine, University of Freiburg, Freiburg, Germany; 10 CIBSS-Center for Integrative Biological Signaling Studies, University of Freiburg, Freiburg, Germany; 11 Center for Basics in NeuroModulation (NeuroModulBasics), Faculty of Medicine, University of Freiburg, Freiburg, Germany; 12 Universitätsmedizin Berlin, Corporate Member of Freie Universität Berlin, Humboldt-Universität zu Berlin, and Berlin Institute of Health, Institute of Pathology, Berlin, Germany; 13 Institute of Experimental Hematology, Center for Translational Cancer Research (TranslaTUM), School of Medicine, Technical University of Munich, Munich, Germany; 14 MRC Molecular Hematology Unit, MRC Weatherall Institute of Molecular Medicine, John Radcliffe Hospital, University of Oxford, Oxford, UK; 15 Institute of Pathology, Ludwig-Maximilians-University, Munich, Germany; 16 Berlin Institute for the Foundation of Learning and Data (BIFOLD) and Charité Universitätsmedizin Berlin, Berlin, Germany; 17 Institute for Immunology, Universitätsklinikum Heidelberg, Heidelberg, Germany; 18 Aix Marseille University, CNRS, INSERM, Centre d’Immunologie de Marseille-Luminy, Marseille, France; 19 Würzburg Institute of Systems Immunology, Max Planck Research Group at the Julius-Maximilians-Universität Würzburg, Würzburg, Germany; 20 Helmholtz Institute for RNA-Based Infection Research (HIRI), Helmholtz Centre for infection Research (HZI), Würzburg, Germany; 21 Present Address: Friedrich Miescher Institute for Biomedical Research (FMI), Basel, Switzerland

**Keywords:** Imaging the immune system, Innate immune cells, Cell migration

## Abstract

Immune cell locomotion is associated with amoeboid migration, a flexible mode of movement, which depends on rapid cycles of actin polymerization and actomyosin contraction^1^. Many immune cells do not necessarily require integrins, the major family of adhesion receptors in mammals, to move productively through three-dimensional tissue spaces^2,3^. Instead, they can use alternative strategies to transmit their actin-driven forces to the substrate, explaining their migratory adaptation to changing external environments^4–6^. However, whether these generalized concepts apply to all immune cells is unclear. Here, we show that the movement of mast cells (immune cells with important roles during allergy and anaphylaxis) differs fundamentally from the widely applied paradigm of interstitial immune cell migration. We identify a crucial role for integrin-dependent adhesion in controlling mast cell movement and localization to anatomical niches rich in KIT ligand, the major mast cell growth and survival factor. Our findings show that substrate-dependent haptokinesis is an important mechanism for the tissue organization of resident immune cells.

## Abstract (web summary)

Immune cells are generally considered to be able to move through tissues using nonadhesive amoeboid migration mechanics. Here, the authors show that, unlike other immune cells, mast cells do not use this method and instead are completely reliant on integrin–ECM interactions.

## Main

**Fig. 1:**
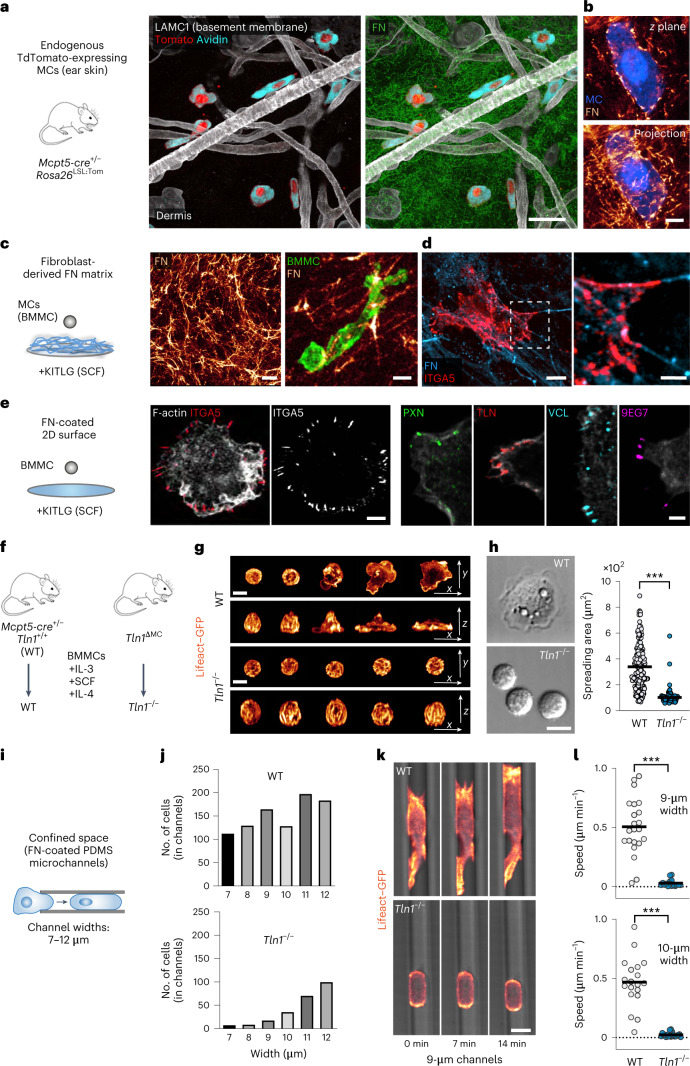
MCs use integrin-dependent force coupling for adhesion to ECM and migration in microchannels. **a**,**b**, Immunofluorescence staining of ear skin whole-mount tissue from an adult *Mcpt5-cre*^+/–^ *Rosa26*^LSL:Tom^ mouse. **a**, Tomato-expressing (red) and avidin^+^ (cyan) MCs in relation to LAMC1^+^ basement membrane components (white) and FN in the dermis. **b**, A Tomato-expressing (blue) interstitial MC enwrapped by dermal FN fibers (glow). A 3-µm projection is shown. **c**, Interactions between living Lifeact–GFP-expressing BMMCs (green) and a fibroblast-derived FN network in vitro. **d**, Fixed BMMCs immunostained for integrin α_5_ (red) display adhesion structures along FN fibers. **e**, Interactions between BMMCs and FN-coated glass slides in the presence of KITLG. Fixed BMMCs were stained for the focal adhesion components integrin α_5_, paxillin, pan-TLN, vinculin and active integrin β_1_ (9EG7). **f**, Scheme illustrating the generation of WT and *Tln1*^−/−^ BMMCs. **g**,**h**, MC spreading on FN-coated 2D surfaces in response to IgE/DNP–HSA. **g**, Lifeact–GFP-expressing WT and *Tln1*^−/−^ BMMCs. A time sequence over 20 min after DNP–HSA stimulation was acquired using live-cell microscopy. Merged projections of confocal *z* stacks are shown. **h**, Differential interference contrast images of WT and *Tln1*^−/−^ BMMCs at 30 min. Analysis of the spreading area was performed after 1 h. Dots represent values of individual cells (*n* = 189 (WT) and 92 (*Tln1*^−/−^) BMMCs) from one of four independent experiments for each genotype. Bars display the mean; ***P < 0.0001. Data were analyzed by two-sided *U*-test. **i**, BMMC migration in the confined space of PDMS microchannels. **j**, WT and *Tln1*^−/−^ BMMC invasion into channels of varying widths was quantified. Data from one multichannel experiment per genotype are displayed. **k**, Lifeact–GFP-expressing (glow) WT and *Tln1*^−/−^ BMMCs migrating in 9-µm-wide channels. A time sequence over 14 min was acquired using live-cell imaging. **l**, Average cell speeds of individual cells in channels 9 µm (*n* = 22 (WT) and 16 (*Tln1*^−/−^) BMMCs) and 10 µm (*n* = 19 (WT) and 20 (*Tln1*^−/−^) BMMCs) wide from one multichannel experiment per genotype are shown. Bars display the mean; ***P < 0.0001. Data were analyzed by two-sided *U*-test. Images were acquired using confocal fluorescence microscopy (**a**, **b**, **d** and **e**) or spinning-disk confocal microscopy (**c**, **g** and **k**); scale bars, 30 µm (**a** and **c**, left), 5 µm (**b** and **c**, right; **d**, left; **e**, left), 2 µm (**e**, right), 2.5 µm (**d**, right) and 10 µm (**g**, **h** and **k**). Source data

**Extended Data Fig. 1:**
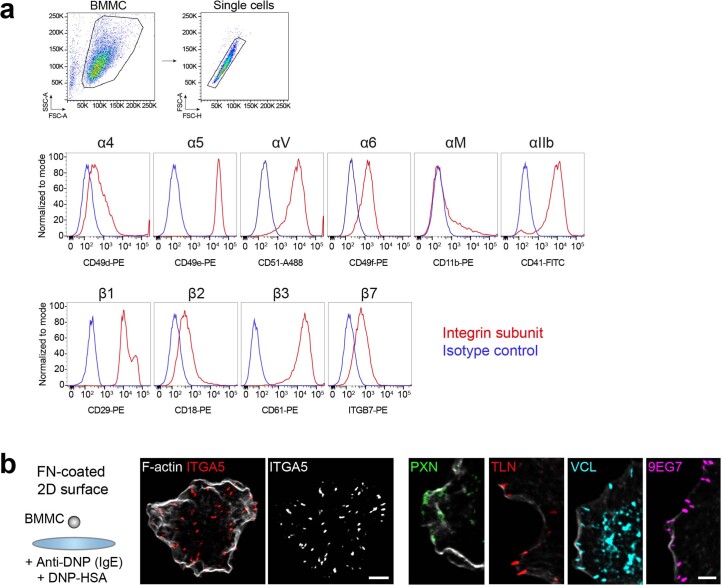
WT BMMCs express several integrin receptors and form β1 integrin-containing adhesion structures on FN-coated 2D surfaces. **(a)** BMMCs that were generated in the presence of IL-3, KITLG and IL-4 over 6 weeks were analyzed by flow cytometry for the cell surface expression of α and β integrin subunits. Isotype control stainings are displayed in blue, integrin subunit-specific stainings in red. **(b)** Interaction of BMMCs with FN-coated glass slides 30 min after IgE/DNP–HSA-induced cell spreading. Confocal fluorescence microscopy of BMMCs stained for F-actin and the focal adhesion components integrin α5, paxillin (PXN), pan-talin (TLN), vinculin (VCL) and active integrin β1 (9EG7). Representative images of *n* = 10 cells from one of two independent experiments. Scale bars: 5 µm (b left), 2 µm (b right).

Mast cells (MCs) are best known in the context of allergy and anaphylaxis but are also critically involved in the degradation of toxins and immunity against various types of pathogens^7,8^. MCs are long-lived immune cells that distribute as resident cellular networks throughout tissues that interact with the external environment. MCs in the skin connective tissue of adult mice originate from definitive hematopoiesis and maintain themselves independently from the bone marrow^9^. Their differentiation and survival critically depend on KIT ligand (KITLG; also known as stem cell factor (SCF)), which signals through the receptor tyrosine kinase KIT^10^. However, it remains unknown how MCs move and strategically position to maintain their survival and cellular network distribution in physiological tissues. Although mouse and human MCs are known to express integrin receptor classes that could interact with other cells or extracellular matrix (ECM)^11^, it has remained unanswered whether and how these adhesion receptors control MC migration, positioning and organization in the native tissue environment. Previous work found MCs in the fibrillar interstitial space and closely aligned to blood vessels and nerves^12^. Visual examination of endogenous MCs in the ear dermis of *Mcpt5-cre*^+/–^ *Rosa26*^LSL:Tom^ mice confirmed these findings. Moreover, the close proximity of MCs to vascular basement membranes and to fibrillar fibronectin (FN) in the interstitial space suggests functional contact between MCs and the ECM (Fig. 1a,b). To investigate the nature of MC–ECM interactions, we used bone marrow-derived MCs (BMMCs) that were differentiated toward a connective tissue-type MC (CTMC) phenotype. These primary MCs highly express ECM-binding integrin heterodimers of the β_1_ and α_v_ family (Extended Data Fig. 1a). When placed on fibroblast-derived FN matrices, BMMCs formed integrin α_5_β_1_-containing adhesion structures reminiscent of fibrillar adhesions in fibroblasts (Fig. 1c,d)^13^. BMMCs also spread on FN-coated two-dimensional (2D) surfaces in the presence of KITLG (Fig. 1e) or after FcεR1 engagement (Extended Data Fig. 1b)^14,15^. Under both conditions, BMMCs formed focal adhesion structures, which were detected based on the presence of integrin α_5_β_1_, paxillin, talin (TLN), vinculin and activated β_1_ integrin (9EG7) predominantly at the cell periphery (Fig. 1e and Extended Data Fig. 1b). Thus, MCs form prominent integrin-dependent adhesion structures when interacting with several forms of ECM.

**Extended Data Fig. 2:**
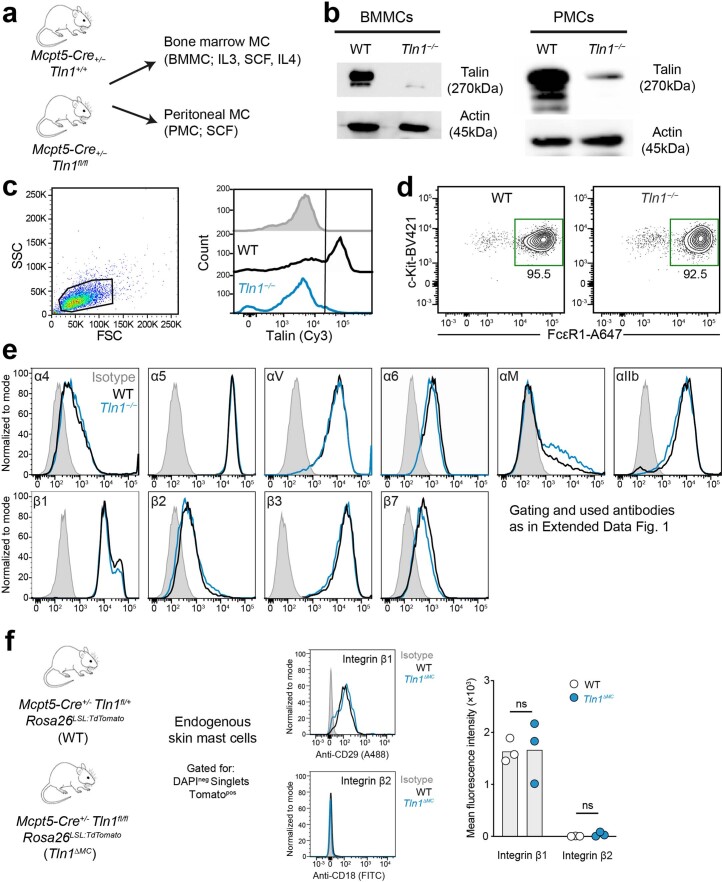
Normal maturation and integrin cell surface expression of MCs upon TLN-1 depletion. **(a)** Scheme for mouse strains and procedures to analyze TLN-1 function in several types of MCs, including bone marrow-derived MCs (BMMCs, cultured with additional IL-4) and peritoneal MCs (PMCs). **(b, c)** The efficiency of conditional TLN-1 knockout was confirmed by immunoblot analysis (b) and flow cytometry (c) by using a pan-talin antibody (recognizing TLN-1 and TLN-2). Cell lysates were generated from BMMC or PMC cultures of WT and *Mcpt5-Cre*^*+/−*^ *Tln1*^*fl/fl*^ mice. Actin was used as loading control. Representative images of two biological replicates (b). Intracellular flow cytometry of BMMCs, gated by FSC/SSC, confirmed the results from the immunoblot. Secondary antibody control is shown on top (grey filled). **(d)** Normal expression of the MC maturation markers c-KIT and FcεR1 in BMMC cultures of WT and *Mcpt5-Cre*^*+/−*^ *Tln1*^*fl/fl*^ mice. Gating for BMMC by FSC/SSC in (d) was done as in (c). **(e)** Normal cell surface expression of integrin subunits on *Tln1*^*−/−*^ BMMCs (in comparison to WT BMMCs, dataset from Extended Data Fig. 1a). **(f)** Normal β1 and β2 integrin cell surface expression in endogenous skin MCs of *Mcpt5-Cre*^*+/−*^ *Tln1*^*fl/fl*^ *Rosa26*^*LSL:TdTomato*^ (*Tln1*^*ΔMC*^) mice in comparison to MCs from *Mcpt5-Cre*^*+/−*^ *Tln1*^*+/fl*^ *Rosa26*^*LSL:TdTomato*^ (WT) mice. Quantification of flow cytometric measurements for integrin expression was based on *n* (WT) = 3 and *n* (*Tln1*^*ΔMC*^) *=* 3 mice. Bars display the mean; *P* = 0.9361 (β1), *P* = 0.2217 (β2), ns, non-significant, two-sided *t* tests. Source data

**Extended Data Fig. 3:**
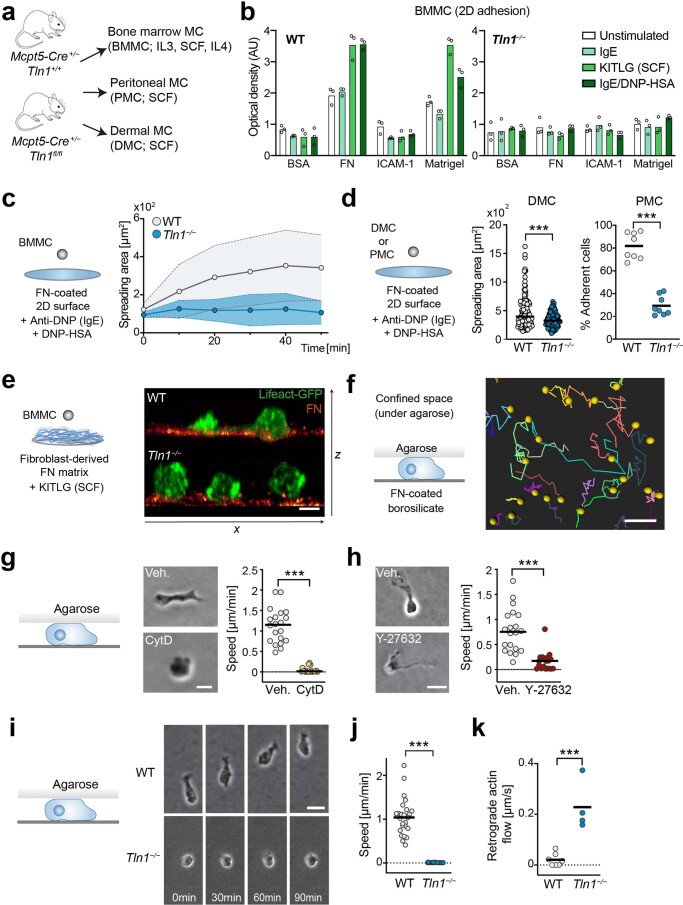
High-affinity integrins are required for MC adhesion on 2D surfaces, cell-derived matrix and for MC migration in confined spaces. **(a)** Analysis of TLN-1 function in several MC types. **(b)** Adhesion assay to several integrin ligands upon IgE, KITLG or IgE/DNP–HSA stimulation. Bar charts are average optical density (OD) values of adherent cells. Data are from one representative (*n* = 3 technical replicates, mean ± s.d.) of three independent experiments. **(c)** MC spreading on FN-coated 2D surfaces upon IgE/DNP–HSA. Dots display mean ± s.d. from one representative experiment. **(d)** MC spreading on FN-coated 2D surfaces upon IgE/DNP–HSA. For DMCs, spreading areas after one hour are compared. Dots represent individual cells (*n* = 137, 141) from two independent experiments. Bars display the mean; ***P = 0.0001, two-sided *U* test. For PMCs, the percentage of adhesive cells was calculated 30 min after stimulation. Dots represent imaging field of views of separate culture wells (*n* = 8, 8) obtained from two independent experiments. ***P < 0.0001, two-sided *t* test. **(e)** Spinning disk-confocal microscopy of Lifeact–GFP expressing BMMCs interacting with fibroblast-derived fibronectin matrix. Side view of a confocal *z*-stack. **(f)** Live imaging of BMMC migration (under-agarose assay). WT cell trajectories over 14 hours. **(g,h)** Contribution of actin polymerization (g, cytochalasin D) or actomyosin contraction (h, Y-27632) for BMMC migration (under-agarose assay). Morphologies and the average cell speed from one experiment per condition. Dots represent individual cells (*n* = 20 randomly chosen cells per condition). Bars display the mean; ***P < 0.0001, two-sided *U* tests. **(i)** BMMC morphologies and movement over 90 min. **(j)** Average cell speeds from one representative experiment of *n* = 3 independent experiments for each genotype are shown. Dots represent individual cells (*n* = 25 randomly chosen cells per genotype). Bars display the median; ***P < 0.0001, two-sided *U* test. **(k)** Actin dynamics of Lifeact–GFP expressing BMMCs were recorded with TIRF microscopy and retrograde actin flow calculated. Each dot represents one cell (*n* = 7, 4). Bars display the mean; ***P = 0.0004, two-sided *t* test. Scale bars: 10 µm (e), 100 µm (f), 15 µm (g), 30 µm (h,i). Source data

To interfere with integrin-based adhesion, we generated TLN-1-deficient BMMCs from *Mcpt5-cre*^+/−^ *Tln1*^fl/fl^ (*Tln1*^ΔMC^) mice (Fig. 1f and Extended Data Fig. 2a). TLN-1 interacts with integrin cytoplasmic domains and is crucial for integrin activation, ligand binding and coupling of F-actin to adhesion sites^16^. As hematopoietic cells express only low levels of the TLN-2 isoform, our experimental strategy efficiently reduced pan-TLN protein levels in MCs (Extended Data Fig. 2b,c) without altering MC maturation (Extended Data Fig. 2d) and integrin cell surface expression (Extended Data Fig. 2e,f). As expected, *Tln1*^−/−^ BMMCs did not bind to ECM substrates, which wild-type (WT) BMMCs adhered to under several experimental conditions (Extended Data Fig. 3a,b). Live-cell imaging (Fig. 1g and Supplementary Video 1) and 2D spreading analysis (Fig. 1h and Extended Data Fig. 3c) corroborated this adhesion deficit. Adhesion responses of *Tln1*^−/−^ dermal MCs and peritoneal MCs were also impaired (Extended Data Fig. 3d). Consequently, *Tln1*^−/−^ BMMCs could not invade fibroblast-derived FN matrices (Extended Data Fig. 3e and Supplementary Video 2). Next, we tested the migration potential of *Tln1*^−/−^ BMMCs in confined spaces, which allow specific immune and non-immune cell types to switch from adhesion-dependent to adhesion-free migration modes^17,18^. In the under-agarose assay (Extended Data Fig. 3f), live imaging over 14 h revealed that WT BMMCs perform lamellipodia-based migration, which depended on F-actin dynamics (Extended Data Fig. 3g) and actomyosin contraction (Extended Data Fig. 3h). By contrast, *Tln1*^−/−^ BMMCs formed only rudimentary cell protrusions and remained largely immotile due to insufficient coupling of actin flow to the substrate (Extended Data Fig. 3i–k and Supplementary Video 3 and 4). When studying MC migration in microchannels of varying widths (Fig. 1i), we observed that TLN-1 deficiency impairs MC invasion into the channels (Fig. 1j). In addition, *Tln1*^−/−^ cells that had managed to enter the confined space showed stalled movement (Fig. 1k,l and Supplementary Video 5). Thus, MCs use integrin-dependent force coupling to ECM for adhesion and movement on 2D surfaces and cell-derived matrices and in confined environments.

**Fig. 2:**
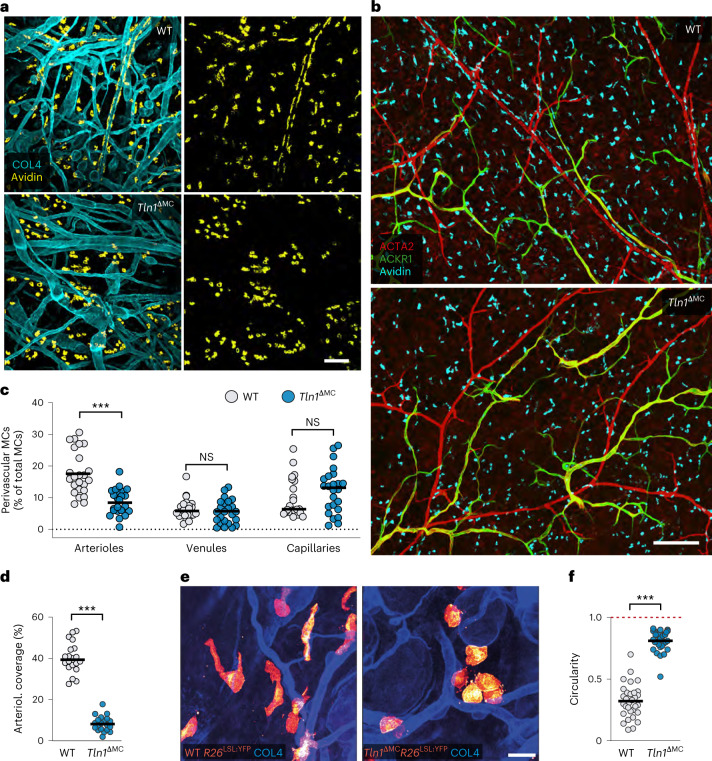
Integrins control MC network formation and periarteriolar alignment in vivo. **a**,**b**, Comparative analysis of ear skin whole-mount tissues of adult *Tln1*^ΔMC^ mice and littermate controls. Endogenous dermal MCs were immunostained with avidin in relation to collagen IV (COL4)-expressing basement membrane structures (**a**) or dermal vessel types (**b**). Arterioles (red) and postcapillary venules (green) were classified by differential staining for α-smooth muscle actin (ACTA2) and ACKR1. Representative micrographs of 20 mice per genotype (**a**) and 5 mice per genotype (**b**) are displayed. **c**,**d**, Analysis of perivascular MC positioning. **c**, Quantification of MCs in proximity to arterioles, venules and capillaries, which were identified by differential staining for ACTA2 and endomucin. Dots represent individual imaging fields of view (*n* = 24 (WT) and 24 (*Tln1*^ΔMC^)), which came from six 7-week-old mice (arterioles: ***P < 0.0001, two-sided *t-*test; venules: *P* = 0.47, two-sided *U*-test; capillaries: *P* = 0.15, two-sided *U-*test); NS, not significant. **d**, Quantification of arteriolar coverage by MCs. Dots represent individual imaging fields of view (*n* = 20 (WT) and 20 (*Tln1*^ΔMC^)) pooled from five 7-week-old mice; ***P < 0.0001. Data were analyzed by two-sided *t*-test. **e**,**f**, Analysis of MC morphologies in the dermis of adult *Tln1*^ΔMC^ *Rosa26*^LSL:YFP^ mice and littermate control *Mcpt5-cre*^+/−^ *Tln1*^+/+^ *Rosa26*^LSL:YFP^ mice. **e**, YFP-expressing dermal MCs are displayed in glow heat map (**e**). Quantification of cell roundness (circularity) was performed for *n* = 31 cells per genotype, which came from four WT and three *Tln1*^ΔMC^ mice (**f**). Bars display the mean; ***P < 0.0001. Data were analyzed by two-sided *U-*test; scale bars, 100 µm (**a**), 200 µm (**b**) and 20 µm (**e**). Source data

**Extended Data Fig. 4:**
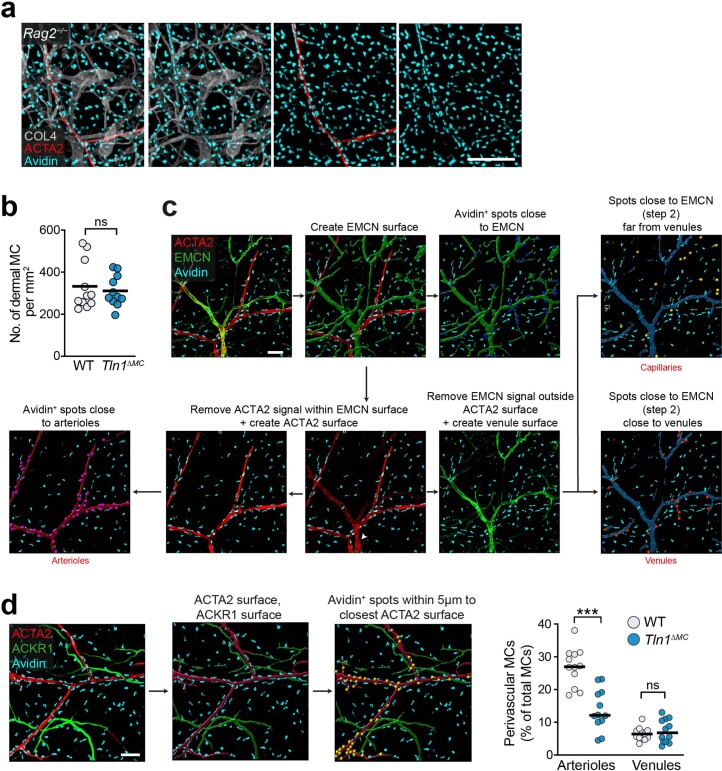
Analysis of MC network formation and periarteriolar alignment *in vivo*. (**a)** Homogenous MC distribution and periarteriolar MC alignment in the dermis of IgE-deficient *Rag2*^*−/−*^ mice. Representative ear skin whole mount immunofluorescence staining of an 8-week old adult mouse of *n* = 3 mice: collagen IV (white), avidin (cyan), ACTA2 (red). **(b)** Histological quantification of MC density in the dermis of ear skin whole mount tissue. Each dot represents the average value of four imaging field of views from one mouse (*n* = 11 mice per genotype); *P* = 0.6026, ns, non-significant, two-sided *t* test. Bars display the mean. **(c,d)** Two experimental strategies to identify dermal vessel types were applied for the analysis of MC proximity to different blood vessels. (c) Workflow for the post-imaging analysis of MC proximity to arterioles, venules and capillaries with Imaris software. This analysis was based on immunofluorescence stainings of ear skin whole mount for MCs (avidin), α-smooth muscle actin (ACTA2) and endomucin (EMCN). ACTA2^+^/EMCN^−^ vessels were classified as arterioles, ACTA2^+^/EMCN^+^ vessels as venules and ACTA2^−^/EMCN^+^ vessels as capillaries. This workflow was used for the data analysis in Fig. 2c. (d) Workflow for the post-imaging analysis of MC proximity to arterioles, venules and capillaries with Imaris software. This analysis was based on immunofluorescence stainings of ear skin whole mount for MCs (avidin), α-smooth muscle actin (ACTA2) and ACKR1. ACTA2^+^/ACKR1^−^ vessels were classified as arterioles and ACTA2^+^/ACKR1^+^ vessels as postcapillary venules (see Fig. 2b). MC proximity analysis was comparable to the results obtained with ACTA2/EMCN stainings in Fig. 2c. Dots represent individual imaging fields of view (*n* = 12, 12), which come from five adult mice; *** *P* < 0.0001 (Art.), *P* = 0.4370, ns (Ven.); two-sided *t* tests. Bars display the mean. Scale bars: 300 µm (a), 100 µm (c,d). Source data

**Extended Data Fig. 5:**
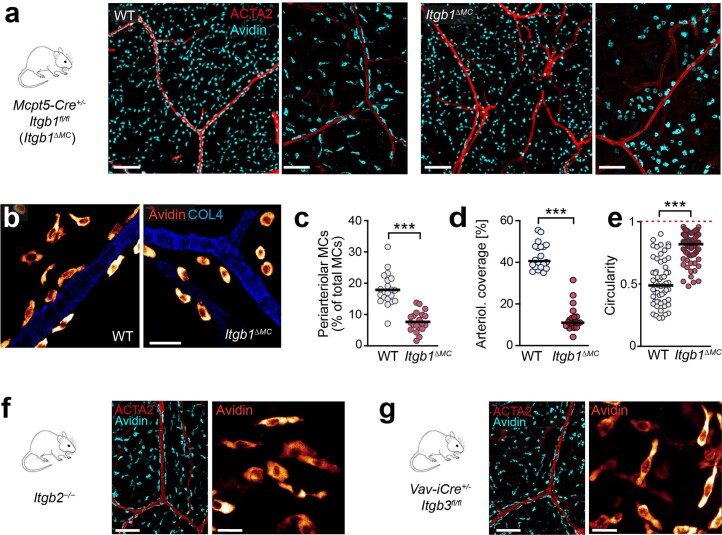
MC-specific depletion of β1 integrins resembles the phenotype of *Tln1*^*ΔMC*^ mice. **(a)** Representative overview of MC shape and distribution in ear skin whole mount immunofluorescence stainings of adult *Mcpt5-Cre*^*+/−*^ *Itgb1*^*fl/fl*^ (*Itgb1*^*ΔMC*^) and littermate control mice. Images are from one of *n* = 7 mice for each genotype. **(b-e)** Comparative analysis of MC shape and distribution in *Itgb1*^*ΔMC*^ and littermate control mice. (b) MC morphologies in native ear dermis of WT and *Itgb1*^*ΔMC*^ mice, (c) quantification of arteriolar MC numbers, (d) arteriolar coverage and (e) circularity in comparison to littermate control mice. For both (c) and (d), dots represent individual imaging fields of view (*n* = 20 per genotype), which come from five mice per genotype; ***P < 0.0001, two-sided *t* test (c), ***P < 0.0001, two-sided *U* test (d). (e) Quantification of cell roundness (circularity) was performed for *n* (WT) = 59 cells and *n* (*Itgb1*^*ΔMC*^)=91 cells, which came from three mice per genotype. Bars display the median; ***P < 0.0001, two-sided *U* test. **(f,g)** Representative images of MC distribution and morphologies in native ear dermis of (f) *Itgb2*^*−/−*^ *(n* = *3)* and (g) *Vav-iCre*^*+/−*^ *Itgb3*^*fl/fl*^ (*n* = 2) mice. Scale bars: 200 µm (a, overview), 100 µm (a, right panels of genotype, f left, g left), 30 µm (b), 20 µm (f right, g right). Source data

To understand the role of integrins in MC movement in real tissues, we investigated the homeostatic ear skin of adult mice, where the residing MCs organize as networks of individual cells that distribute widely throughout the dermal connective tissue. We used *Tln1*^ΔMC^ mice with specific deletion of *Tln1* in endogenous CTMCs. Comparison of dermal MC distribution between *Tln1*^ΔMC^ and control animals revealed two striking phenotypes. First, TLN-1-deficient MCs lost their normal tissue distribution and instead formed cellular clusters in the dermis (Fig. 2a,b). This was not observed in *Rag2*^−/−^ mice, ruling out a role of IgE-stimulated MC adhesion in this process (Extended Data Fig. 4a)^15^. Dermal MC numbers were comparable between *Tln1*^ΔMC^ and control mice (Extended Data Fig. 4b). Second, TLN-1-deficient MCs lost their localization along dermal arterioles (Fig. 2b). Perivascular alignment in *Tln1*^ΔMC^ mice was only affected at arterioles but not at postcapillary venules or capillaries (Fig. 2b,c and Extended Data Fig. 4c,d). Consequently, MC coverage of the outer arteriolar walls was diminished in knockout animals (Fig. 2d). Cell shape analysis of yellow fluorescent protein (YFP)-expressing CTMCs revealed obvious differences. WT MCs displayed elongated, mesenchymal morphologies with lamellopodial-like leading edges, whereas TLN-1-deficient MCs showed round, amoeboid-like shapes (Fig. 2e,f). MC-specific depletion of β_1_ integrins in *Mcpt5-cre*^+/−^ *Itgb1*^fl/fl^ mice also displayed a mesenchymal-to-amoeboid shape switch and similar decreases in the percentage of periarteriolar MC and arteriolar MC coverage as *Tln1*^ΔMC^ mice (Extended Data Fig. 5a–e). These phenotypes were not observed in *Itgb2*^−/−^ and *Vav-iCre Itgb3*^fl/fl^ mice (Extended Data Fig. 5f,g). Thus, ECM-binding β_1_ integrins have crucial roles for maintaining MC shape, positioning and organization in living connective tissue.

**Fig. 3:**
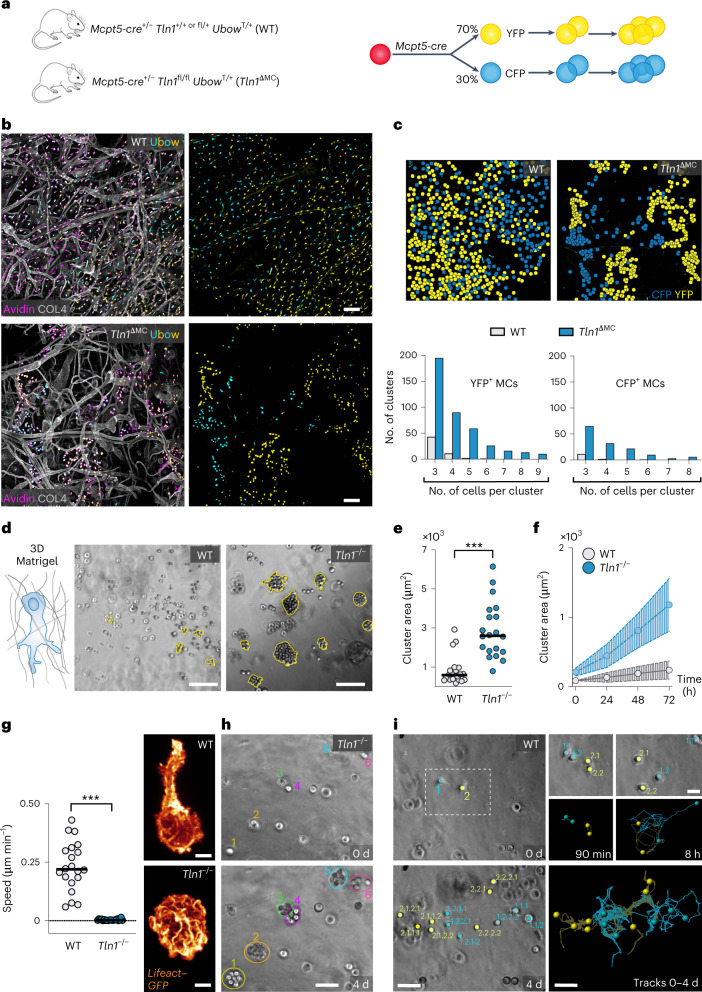
MCs use slow integrin-dependent migration in tissues and 3D matrix. **a**, Tracing slow MC migration in the dermal tissue of 6- to 12-week-old mice. *Tln1*^ΔMC^ and control mice carried the Ubow reporter transgene. **b**, Comparative analysis of ear skin whole-mount tissue of 8-week-old WT Ubow and *Tln1*^ΔMC^ Ubow mice. All YFP^+^ and CFP^+^ cells were positive for the pan-MC marker avidin (purple). Dermal tissue was counterstained with collagen IV (white) to assess basement membranes and overall tissue geometries. **c**, Top, representative images of cell center coordinates after computational rendering using ClusterQuant2D software, which was used to analyze the formation and sizes of unicolored MC clusters. Bottom, quantification of YFP^+^ and CFP^+^ cluster frequencies sorted by cluster size. Two-cell clusters were not included in the analysis. Data are from *n* = 3 (WT) and *n* = 4 (*Tln1*^ΔMC^) mice with two to three imaging fields of view per mouse. **d**–**f**, WT and *Tln1*^−/−^ BMMCs in 3D Matrigel were monitored over 3 d. **d**, Brightfield images at *t* = 72 h. **e**, Quantification of the cell cluster area at *t* = 72 h. Each dot represents the growth area of one MC cluster (*n* = 23 (WT) and 20 (*Tln1*^ΔMC^)) obtained from three independent experiments. Bars display the medians; ***P < 0.0001. Data were analyzed by two-sided *U*-test. **f**, Analysis of MC cluster growth area over time. Dots show individual clusters (mean ± s.d.; *n* = 10 clusters from one experiment). **g**–**i**, BMMC migration in 3D Matrigel was recorded by live-cell microscopy over 4 d. **g**, Quantification of the average cell speed over the first 36 h. Dots are values of individual cells (*n* = 20 randomly chosen cells per genotype). Bars display the means; ***P < 0.0001. Data were analyzed by two-sided *U*-test. Images were acquired by spinning-disk confocal microscopy of Lifeact–GFP-expressing WT and *Tln1*^−/−^ BMMCs migrating in Matrigel. **h**, Proliferation of individual *Tln1*^−/−^ BMMCs (colored numbers) but hardly any movement out of cell clusters. **i**, Tracking analysis revealed intermittent phases of movement and cell division, which distributes WT cells in the gel. Colored numbers indicate parent and descendant cells; scale bars, 100 µm (**b** and **d**), 5 µm (**g**), 50 µm (**h** and **i**) and 20 µm (**i**, inset). Source data

**Extended Data Fig. 6:**
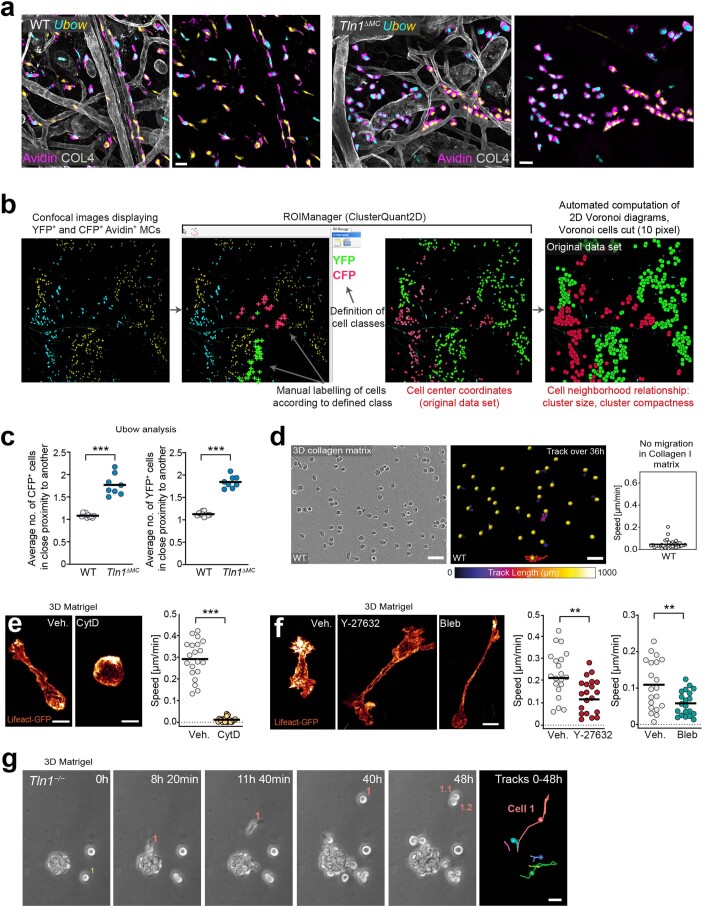
MCs use integrin-dependent migration in tissues and 3D matrigel. **(a)** Dermal MC shape and distribution in adult WT and *Tln1*^*ΔMC*^ Ubow mice (Fig. 3a), stained with pan-MC marker avidin (purple) and anti-collagen IV (white). Representative images from one of *n* = 6 mice per genotype. **(b)** Workflow for the analysis of MC cluster formation with ClusterQuant2D software. **(c)** Average YFP^+^ and CFP^+^ cluster size in WT Ubow or *Tln1*^*ΔMC*^ Ubow mice. A value of 1 indicates that no cell sits in close proximity to another cell (homogeneous distribution). A value > 1 indicates that fractions of cells sit in close proximity to another and form clusters of > 1 cell. Dots represent imaging fields of view (*n* (WT) = 10, *n* (*Tln1*^*ΔMC*^) = 8) from three WT and four *Tln1*^*ΔMC*^ mice. Bars display the mean; ***P < 0.0001, two-sided *t* tests. **(d)** WT BMMCs lack mesenchymal-like morphology and migration in 3D collagen I matrices. Live imaging over 36 h and quantification of average cell speeds. Dots are individual cells (*n* = 30 randomly chosen cells). **(e,f)** Contribution of actin polymerization (e, cytochalasin D) and actomyosin contraction (f, Y-27632 or blebbistatin) for BMMC migration in 3D matrigel. Spinning-disk images of Lifeact–GFP expressing WT cells in 3D gels. Average cell speeds over 36 h from one experiment per genotype. Dots are individual cells (*n* = 20 randomly chosen cells per condition). Bars display the mean; ***P < 0.0001, two-sided *t* test (e). **P = 0.0019 (Y-27), ** *P* = 0.0046 (Bleb), two-sided *t* tests (f). **(g)** Example of a proliferating *Tln1*^*−/−*^ BMMC cluster in 3D matrigel, live cell microscopy over 48 h. Rarely, individual cells move away from proliferating clusters, forming seeds for a new cluster (for example Cell 1 moves, then divides to form descendant cells 1.1 and 1.2). Tracks over 48 h are shown. Example sequence from two independent experiments. Scale bars: 25 µm (a), 10 µm (e,f), 30 µm (g). Source data

Dermal MCs are long-lived cells with slow proliferation rates and maintain themselves during homeostasis in adult mice^9^. Intravital imaging studies revealed extremely slow, if any, MC kinetics over several hours^19,20^. We hypothesized that the observed MC clusters in *Tln1*^ΔMC^ mice resulted from an MC migration deficit (Fig. 2a,b). To address this question, we crossed *Tln1*^ΔMC^ mice with transgenic Ubow mice for in situ cell fate tracking^21^. *Mcpt5* promoter activity drives a Cre recombinase-mediated single and definitive recombination event, by which any Ubow MC acquires a specific color (either YFP or cyan fluorescent protein (CFP) at ~2:1 stochastics) that is stable for the life of the cell and inherited by all its progeny (Fig. 3a). Analysis of adult ear dermis of control Ubow mice revealed a heterogeneous mixed distribution of YFP^+^ and CFP^+^ MCs (Fig. 3b). By contrast, *Tln1*^ΔMC^ Ubow mice showed areas of unicolored MC clusters, strongly suggesting that MCs originated from a few parent MCs in these regions but then did not move further into the interstitial dermal space to intermix with other MCs (Fig. 3b and Extended Data Fig. 6a). Detailed quantification showed that WT MCs distributed predominantly as single cells or two-cell pairs, which we did not categorize as cell clusters, whereas TLN-1-deficient MCs were commonly associated with clusters of greater than three cells (Fig. 3c and Extended Data Fig. 6b,c).

To prove that cluster formation of adhesion-deficient MCs resulted from a migration deficit, we sought to image BMMCs over several days in an in vitro mimic of the dermal interstitial space. Collagen-based three-dimensional (3D) matrices did not support BMMC migration, as mouse BMMCs, similar to human skin MCs^22^, do not adhere to collagen I due to the lack of collagen I-binding integrins (Extended Data Fig. 6d). In 3D Matrigel, BMMCs moved productively, critically depending on F-actin dynamics (Extended Data Fig. 6e), while actomyosin contraction had only a contributing role (Extended Data Fig. 6f). Comparison of WT and *Tln1*^−/−^ BMMC cultures over 4 d matched our in situ observations. WT cells distributed throughout the gel, whereas *Tln1*^−/−^ cells formed MC clusters over time (Fig. 3d–f). Lack of integrin functionality caused *Tln1*^−/−^ BMMCs to adopt round, amoeboid-like shapes, which did not support migration (Fig. 3g). Consequently, proliferating *Tln1*^−/−^ BMMCs underwent cell division but did not move further into the gel, resulting in MC cluster formation (Fig. 3h and Supplementary Video 6). Only in very rare cases did we observe amoeboid-like migrating *Tln1*^−/−^ BMMCs, which, in these few instances, could seed a new MC cluster (Extended Data Fig. 6g and Supplementary Video 7). By contrast, WT BMMCs moved away from each other after division, allowing a few individual parent cells to completely seed the gel matrix over 4 d (Fig. 3i and Supplementary Video 6). Together, we demonstrate that MC migration in physiological 3D environments strictly depends on integrin-mediated force coupling, which is required to organize the resident MC population and distribute individual MCs widely throughout interstitial spaces.

**Fig. 4:**
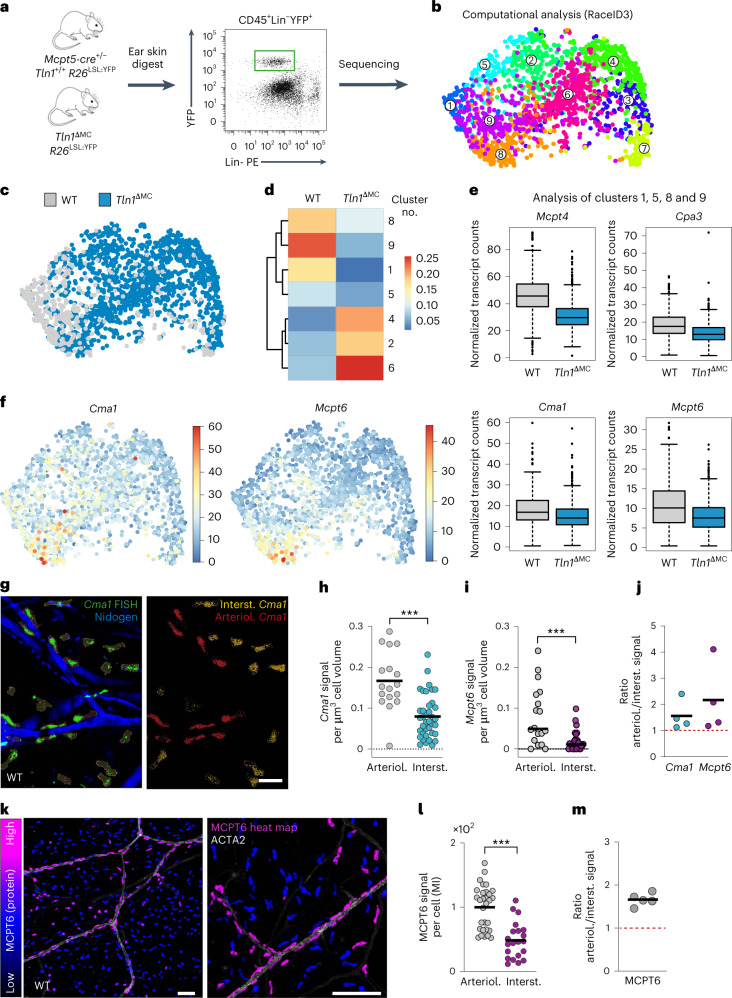
Integrin-dependent adhesion confines a mature MC subset to arterioles. **a**, Workflow for scRNA-seq analysis of MCs from dermal ear skin. **b**, UMAP of single-cell transcriptomes of sorted CD45^+^Lin^−^YFP^+^ cells (WT and *Tln1*^∆MC^ MCs combined, *n* = 1,895 cells derived from three WT and three *Tln1*^∆MC^ mice) highlighting RaceID3 clusters. Numbers denote clusters. **c**, UMAP of single-cell transcriptomes of MCs highlighting the sources or origins of the cells, that is, which mice (WT or *Tln1*^∆MC^) the cells were isolated from. **d**, Heat map showing the fractions of WT and *Tln1*^∆MC^ MCs in clusters, which were significantly enriched for either WT or *Tln1*^∆MC^ cells; *P* < 0.05. Data were analyzed by one-tailed hypergeometric test. **e**, Box plots show analysis of clusters 1, 5, 8 and 9. MC marker expression was downregulated in MCs from *Tln1*^∆MC^ relative to WT mice. Boxes indicate first and third quartiles, and the black line indicates the median. Whiskers indicate 5% and 95% quantiles. For all comparisons, Benjamini–Hochberg-corrected *P* < 0.05; *Mcpt4*, *P* = 2.9 × 10^–31^; *Cpa3*, *P* = 2.5 × 10^–69^; *Cma1*, *P* = 3.8 × 10^–13;^ *Mcpt6*, *P* = 4.1 × 10^–23^. **f**, UMAP for *Cma1* and *Mcpt6* expression. The color bar indicates normalized transcript counts. **g**, FISH of *Cma1* mRNA in ear skin whole mounts of adult WT mice (left) and postimaging analysis of *Cma1* expression in arteriolar (arteriol.) versus interstitial (interst.) MCs (right). **h**–**j**, Quantification of *Cma1* mRNA (**h**) and *Mcpt6* mRNA (**i**) FISH signal per cell volume and comparison between arteriolar and interstitial MCs. Dots represent individual MCs from one of four biological replicates; *n* = 17 (arteriolar MCs) and 38 (interstitial MCs; **h**) and *n* = 18 (arteriolar MCs) and 28 (interstitial MCs; **i**). Bars indicate means; ***P < 0.0001, two-sided *t*-test (**h**); ***P = 0.0009, two-sided *U*-test (**i**). **j**, Ratios of arteriolar to interstitial mRNA signals were calculated for four mice. **k**–**m**, MCPT6 protein expression in dermal MCs of adult WT mice. **k**, Immunofluorescence staining for MCPT6 in ear skin whole-mount tissue. An overview image (left) and MCs in the periarteriolar space (right) are shown. The heat map bar shows MCPT6 fluorescence signal intensity. **l**, Quantification of MCPT6 fluorescence. The mean intensity (MI) of MCPT6 fluorescence was measured per cell in arteriolar versus interstitial MCs. Quantification is displayed for one independent experiment, and each dot represents one MC; *n* = 30 (arteriolar MCs) and 21 (interstitial MCs). Bars indicate means; ***P < 0.0001, two-sided *t*-test. **m**, Ratios of arteriolar to interstitial MCPT6 signal were calculated for five mice; scale bars, 20 µm (**g**), 100 µm (**k**, left) and 150 µm (**k**, right). Source data

**Extended Data Fig. 7:**
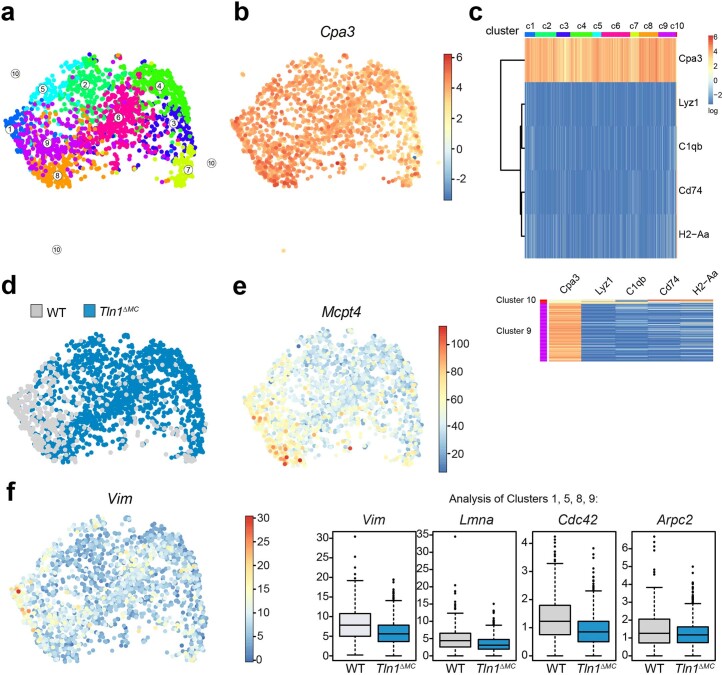
Single cell RNAseq analysis of dermal MCs. **(a)** UMAP of single-cell transcriptomes of sorted CD45^+^Lin^−^YFP^+^ cells (WT and *Tln1*^∆MC^ MCs combined) highlighting RaceID3 clusters, displaying clusters 1–10. The outlying cluster 10 was not displayed in the corresponding Fig. 4b, as it carried an additional macrophage gene signature (see Extended Data Fig. 7c). Numbers denote clusters. *n* = 1895 cells derived from three WT and three *Tln1*^∆MC^ mice). **(b)** UMAP showing the expression of the gene encoding the MC marker CPA3 in clusters 1–10. The color bar indicates log_2_ normalized expression. **(c)** Heatmap showing *Cpa3* expression and genes specifically expressed in cluster 10. The color bar at the top of the heatmap indicates the RaceID3 clusters. Scale bar, log_2_ normalized expression. Below, the heatmap of clusters 10 and 9 are also displayed at higher magnification. **(d)** UMAP of single-cell transcriptomes of MCs highlighting the sources or origins of the cells, that is which mice (WT or *Tln1*^∆MC^) the cells were isolated from. This is a duplicate of Fig. 4c. **(e)** UMAP of *Mcpt4* expression. The color bar indicates normalized transcript counts. **(f)** UMAP of *Vim* expression (left). The color bar indicates normalized transcript counts. Box plots show analysis of clusters 1, 5, 8, 9 and the expression of genes encoding the cytoskeletal elements VIM, LMNA, CDC42 and ARPC2 in WT and *Tln1*^∆MC^ cells (right). The expression of the genes was significantly downregulated in MCs from *Tln1*^∆MC^ relative to WT mice. Boxes indicate 1^st^ and 3^rd^ quartiles and the black line indicates the median. Whiskers indicate 5% and 95% quantiles. For all comparisons: Benjamini-Hochberg corrected *P* < 0.05. *Vim: P* = 4.1e-20, *Lmna: P* = 9.3e-18, *Cdc42: P* = 1e-9, *Arpc2: P* = 0.049.

Next, we performed single-cell RNA sequencing (scRNA-seq) and analysis of endogenous MCs from WT and knockout mice (Fig. 4a,b). Based on the expression of the MC marker *Cpa3*, almost all sorted cells were identified as MCs (Extended Data Fig. 7a–c). Only a very small cell subset showed additional macrophage features (cluster 10; Extended Data Fig. 7c), which we excluded from further analysis. A uniform manifold approximation and projection (UMAP) representation of single-cell transcriptomes of sorted CD45^+^Lin^−^YFP^+^ cells highlighted nine clusters of skin MCs as defined by RaceID3 (ref. ^23^; Fig. 4b). Direct comparison of MCs isolated from WT or *Tln1*^∆MC^ mice highlighted clusters enriched in WT cells (Fig. 4c,d). Closer examination of differentially expressed genes in clusters 1, 5, 8 and 9 revealed two transcriptomic signatures: (1) an upregulation of several genes encoding MC differentiation and maturation markers, including MCPT5 (CMA1), MCPT6, MCPT4 and CPA3 (Fig. 4e,f and Extended Data Fig. 7d,e), and (2) a cytoskeletal signature based on the upregulation of transcripts encoding the intermediate filaments VIM and LMNA and actin regulators CDC42 and ARPC2 (Extended Data Fig. 7f). Thus, integrin-mediated adhesion supports the establishment of a specific MC phenotype in dermal tissue.

**Fig. 5:**
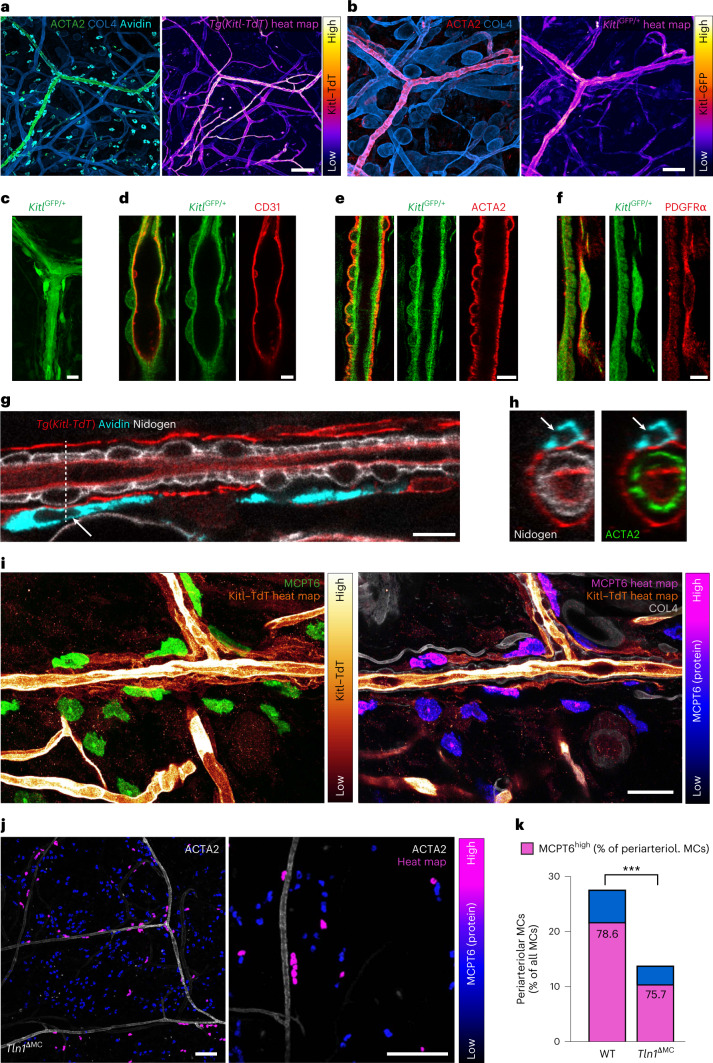
Integrins support MC localization to the KITLG-rich periarteriolar space. **a**,**b**, In situ analysis of *Kitl* promoter activity was performed in the ear dermis of two reporter mouse strains. Ear skin whole mounts of transgenic *Kitl-TdTomato* (*Tg*(*Kitl-TdT*); **a**) and *Kitl*^GFP/+^ (**b**) mice were counterstained for ACTA2-expressing arterioles, COL4^+^ basement membranes and MCs (avidin; only in **a**). Representative examples of *n* = 5 mice are shown. Endogenous fluorescence intensities are also displayed as heat maps. **c**–**f**, Characterization of GFP-expressing stromal cell types along dermal arterioles of *Kitl*^GFP/+^ mice (**c**). Immunofluorescence stainings were performed against CD31 (endothelial cells; **d**), ACTA2 (VSMCs; **e**) and PDGFRα (fibroblasts; **f**). **g**,**h**, Periarteriolar positioning of avidin^+^ MCs (cyan) and interactions with periarteriolar fibroblasts in transgenic *Kitl-TdTomato* mice. A longitudinal view of an arteriole (**g**) and cross-section with arteriolar stromal cell layers (**h**) are shown. White arrows and the dashed line indicate the MC in the cross-section area. **i**, Immunofluorescence analysis of ear skin tissue of transgenic *Kitl-TdTomato* mice reveals close proximity and interaction of MCPT6^high^ cells with periarteriolar KITLG-expressing fibroblasts. Representative images are volume projection (left) and focal *z* plane (right). **j**, Immunofluorescence staining for MCPT6 in ear skin whole-mount tissue of an adult *Tln1*^ΔMC^ mouse. The data presentation is the same as for WT in Fig. 4k. **k**, Comparative analysis of MCPT6 expression in periarteriolar MCs was performed for WT and *Tln1*^ΔMC^ mice. The full bar displays the percentage of periarteriolar MCs of total dermal MCs (similar to Fig. 2c). Data include the analysis of three imaging fields of view collected from five WT and four *Tln1*^ΔMC^ mice (*n* = 15 (WT) and 12 (*Tln1*^*ΔMC*^)); ***P < 0.0001, two-sided *t*-test. Bars also display the percentage of MCPT6^high^-expressing MCs (purple) within this subset of periarteriolar MCs. MCPT6^low^-expressing MCs at arterioles are represented in blue; scale bars, 100 µm (**a** and **j**, left), 50 µm (**b**), 15 µm (**c**), 5 µm (**d** and **f**), 10 µm (**e** and **g**), 30 µm (**i**) and 150 µm (**j**, right). Source data

**Extended Data Fig. 8:**
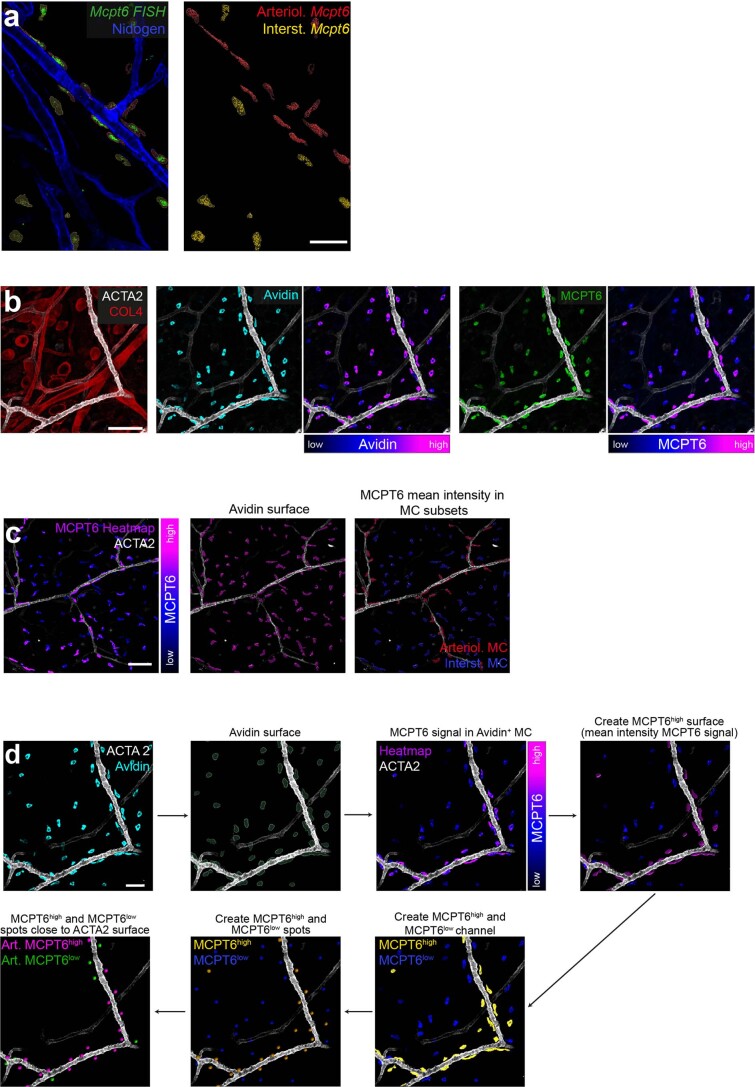
Analysis of the periarteriolar MC phenotype *in situ*. **(a)** Representative visualization of *Mcpt6* mRNA using fluorescence in situ hybridization (FISH) in ear skin whole mount tissue of adult WT mice. These data refer to the post-imaging analysis of *Mcpt6* expression in periarteriolar versus interstitial MCs in Fig. 4i,j. Representative image from one of *n* = 4 mice. **(b)** Differential expression of MCPT6 in periarteriolar versus interstitial MCs. Ear skin whole mount tissue of an adult WT mouse is counter-stained for the pan-MC marker avidin (cyan), MCPT6 (green), COL4-expressing basement membranes (red) and ACTA2-positive arterioles (white). Avidin and MCPT6 fluorescence intensities are also displayed as heatmap. **(c,d)** Imaris software-based workflows for the post-imaging analysis of MCPT6 mean fluorescence intensities in MC subsets (c) and calculation of the percentage of MCPT6^high^ MCs of all periarteriolar MCs in WT and *Tln1*^*ΔMC*^ mice (d). (b-d) Representative images from one experiment of *n* = 5 mice. Scale bars: 50 µm (a, d), and 100 µm (b,c).

**Extended Data Fig. 9:**
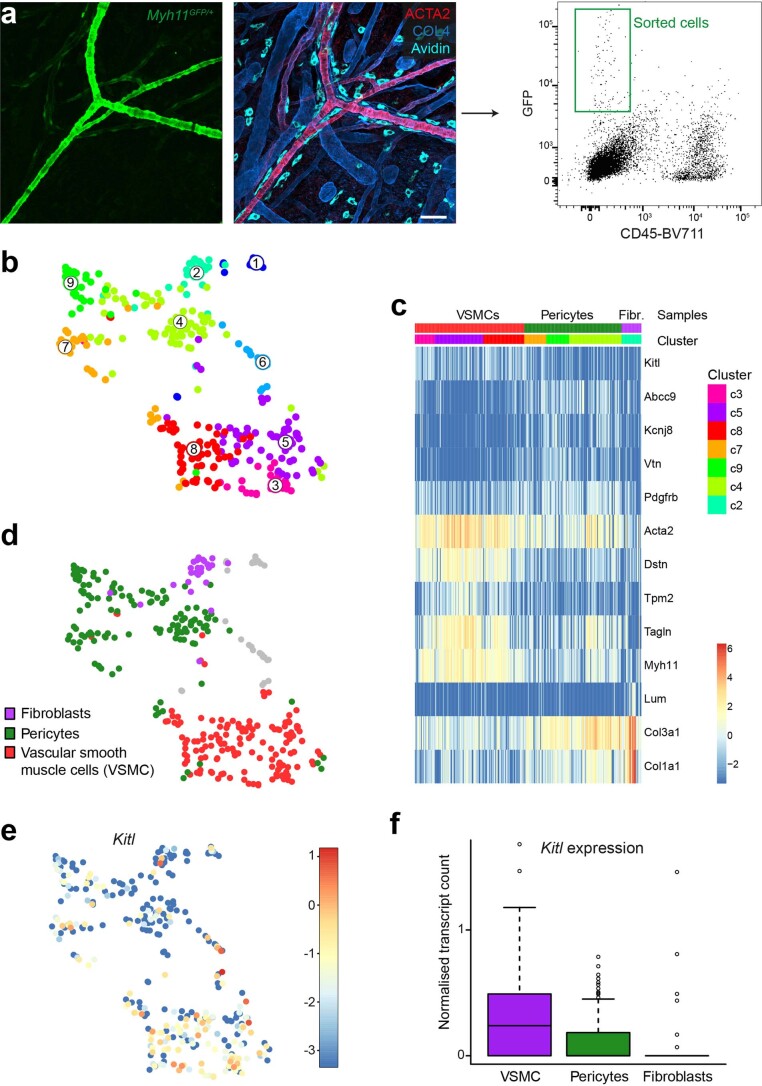
Analysis of periarteriolar stromal cells with scRNAseq. **(a)** Experimental strategy and work flow for scRNAseq analysis of perivascular stromal cells with focus on Myh11–GFP expressing vascular smooth muscle cells (VSMCs). Myh11–GFP transgenic mice carry a strong genetic label for VSMC and very weak GFP-label for pericytes. Ear skin tissue of a Myh11–GFP mouse was digested, and GFP-fluorescent cells sorted in preparation for scRNAseq analysis. Representative image of *n* = 2 mice. **(b)** UMAP highlighting RaceID3 clusters. Numbers denote clusters. *n* = 330 cells. **(c)** Heatmap showing the expression of marker genes for each cell type compartment. Color bars indicate cell type and RaceID3 clusters. Three stromal cell types (VSMC, pericyte, fibroblast) were retrieved after the sort. Scale bar, log_2_ normalized expression. **(d)** UMAP highlighting the annotated stromal cell types. **(e)** UMAP of *Kitl* expression. Color bar indicates log_2_ normalized expression. **(f)** Box plots showing the normalized expression of *Kitl* in VSMCs, pericytes, and fibroblasts. Boxes indicate 1^st^ and 3^rd^ quartiles and the black line indicates the median. Whiskers indicate 5% and 95% quantiles. Scale bars: 50 µm (a).

**Extended Data Fig. 10:**
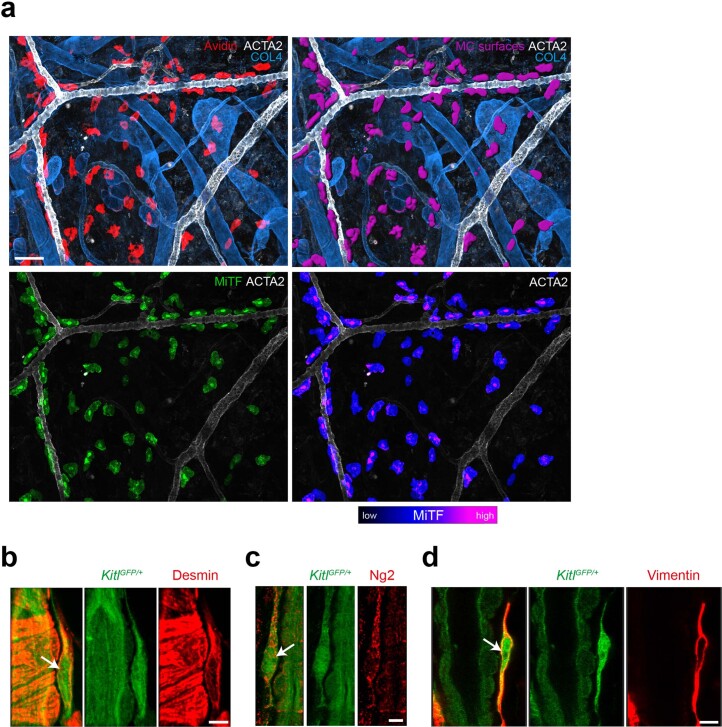
Nuclear MiTF as read-out of KITLG-KIT signaling is detectable in MCs localizing along KITLG-expressing stromal cells in the periarteriolar space. **(a)** Immunofluorescence staining analysis of nuclear microphthalmia-associated transcription factor (MiTF) signal in dermal MCs of ear skin whole mount tissue. MC surfaces (purple) were generated based on avidin staining (red). Only MiTF signals inside MC surfaces are displayed as single color (green) or as heatmap. In contrast to MCs in the interstitial spaces, many of the periarteriolar MCs show strong nuclear MiTF signals, suggesting upregulated KITLG–KIT signaling in MCs localizing in the periarteriolar space. Representative image of *n* = 3 independent experiments. **(b–d)** GFP-expressing fibroblasts that reside along dermal arterioles in *Kitl*^*GFP/+*^ mice were phenotypically characterized by immunofluorescence stainings against desmin (b), chondroitin sulfate proteoglycan (Ng2) (c) and vimentin (d). Representative images of *n* = 4 independent experiments (b–d). Scale bars: 50 µm (a), and 5 µm (b–d).

To characterize phenotypic MC heterogeneity in homeostatic mouse skin, we focused on validating the differential expression of MC marker genes in the intact tissue. To identify MC subsets and relate them to anatomical tissue structures, we performed fluorescence in situ hybridization (FISH) for *Mcpt5* (*Cma1*) and *Mcpt6* in ear skin whole mounts (Fig. 4g and Extended Data Fig. 8a). *Cma1* and *Mcpt6* FISH signals were increased in MCs lining arterioles compared to in MCs in the interstitial space (Fig. 4h–j). This unexpected finding was confirmed by MCPT6 protein detection, which revealed higher MCPT6 signals in MCs lining arterioles in WT mice (Fig. 4k–m and Extended Data Fig. 8b–d). All our imaging analyses suggested that periarteriolar MCPT6^high^ MCs are in direct contact with the ACTA2^+^ vascular smooth muscle cell (VSMC) layer, which surrounds arteriolar endothelial cells (Figs. 2b and 4k and Extended Data Figs. 4c,d and 8b–d)^24^. Hypothesizing that VSMCs might be associated with the mature MC phenotype, we performed scRNA-seq analysis of green fluorescent protein-positive (GFP^+^) cells from ear skin of transgenic *Myh11-GFP* mice. Cells were sorted for GFP^high^-expressing VSMCs, but some GFP^low^ pericytes and a small fraction of fibroblasts were also collected (Extended Data Fig. 9a–d). Computational analysis of the sequencing data revealed higher expression of KITLG (*Kitl*), the major MC growth, survival and differentiation factor, in VSMCs than in other stromal cell types (Extended Data Fig. 9e,f). To confirm this finding in tissue, we analyzed *Kitl* promoter activity in *Kitl*^GFP^ knock-in and transgenic *Kitl-TdTomato* mice. ACTA2^+^ arterioles displayed high fluorescent reporter signal in both mouse strains, revealing strong *Kitl* promoter activity as an indication of KITLG expression in this dermal compartment (Fig. 5a,b). Moreover, periarteriolar MCs displayed the strongest fluorescence signals of nuclear-localized microphthalmia-associated transcription factor, which acts downstream of KITLG–KIT signaling and regulates the expression of key proteins involved in MC differentiation, activity and adhesion (Extended Data Fig. 10a)^25,26^. Detailed analysis of the arteriolar unit showed KITLG expression at the inner lining of CD31^+^ endothelial cells and ACTA2^+^ VSMCs (Fig. 5c–e). Unexpectedly, a periarteriolar single-cell layer of fibroblasts also showed *Kitl* promoter activity (Fig. 5c,f and Extended Data Fig. 10b–d). Periarteriolar MCs located very close to this yet unappreciated subset of dermal fibroblasts (Fig. 5g,h and Supplementary Video 8) and MCs in the direct vicinity of this fibroblast layer were mostly of a mature MCPT6^high^ phenotype (Fig. 5i). In WT mice, ~80% of periarteriolar MCs displayed strong MCPT6 signals (Figs. 4k and 5k). Of the much lower number of MCs interacting with arterioles in *Tln1*^ΔMC^ mice (Fig. 2c), the same percentage of MCs still expressed strong MCPT6 signals (Fig. 5j,k). In other words, the close vicinity of MCs to arterioles, even in the absence of integrin engagement, is sufficient to promote expression of the MC maturation marker MCPT6. Thus, integrin-mediated adhesion has an additional crucial role in supporting MC localization along the arteriolar unit, where several stromal cell types provide a KITLG-rich environment that potentially contributes to the local mature MC phenotype.

In summary, our findings identify MCs as an ECM-anchored immune cell type that critically depends on substrate adhesion for physiological migration but not survival. Their slow haptic mode of movement is perfectly adapted to organize long-lasting positioning in tissues with heterogeneous growth factor distribution. MCs are unique among immune cells in their migration strategy, as they appear fixed to a special mode of interstitial movement in the tissue. While amoeboid-like immune cells, including dendritic cells, neutrophils and lymphocytes, compensate the loss of integrin-dependent adhesion by switching to another mechanistic mode of migration, MCs lack this migratory plasticity. This stands in stark contrast to the rapid and flexible amoeboid migration of many other immune cell types, whose tissue guidance commonly depends on G-protein-coupled receptor signaling^27^. This makes MCs special among immune cells and sets them at the outer end of the immune cell migration spectrum. Our data place MCs instead into the broad category of mesenchymal-like migrating cells despite their hematopoietic origin. However, MCs are not just ‘fibroblast-like’ cells. Lack of integrin functionality does not interfere with MC numbers and the general size of the dermal MC pool. This again contrasts many other mesenchymal cell types, including fibroblasts and endothelial cells, which require β_1_ integrin signaling for anchorage-dependent growth and survival^28,29^. Here, we highlight that integrin-mediated haptokinesis is crucial for distributing individual MCs widely throughout the tissue and organizing the population of long-lived MCs as resident networks. Furthermore, integrin-dependent movement supports site-specific MC positioning to the periarteriolar space where MCs are exposed to high concentrations of KITLG and acquire mature phenotypes. As it has long been known that KITLG–KIT signaling can induce integrin-mediated adhesion in MCs in vitro^30^, we speculate that KITLG may act as a haptotactic cue to recruit adhesive MCs into the periarteriolar space and thus support their tissue localization. However, how exactly KITLG distribution and KIT and integrin receptor signaling cooperate to establish and organize anatomical tissue niches for MC growth, differentiation and homeostasis remains to be investigated. Future studies will also need to formally validate the molecular interactions of periarteriolar mature MCs with KITLG-expressing stromal cells. Together, our study builds the basis for future studies assessing MCs as potential mechanosensitive immune cells, which probably respond to changing tissue properties under pathophysiological conditions of chronic inflammation, fibrosis and tissue trauma.

## Methods

### Mouse models

Mouse breeding and husbandry were performed at the Max Planck Institute of Immunobiology and Epigenetics, Freiburg, in accordance with the guidelines provided by the Federation of European Laboratory Animal Science Association and as approved by German authorities (Regional Council of Freiburg). Mice were only used for organ removal after death by carbon dioxide exposure and thus were not subject to experimental procedures and ethical approval according to §4 (3) Tierschutzgesetz. Mice were maintained in a conventional animal facility with a 14-h light/10-h dark cycle at a temperature of 22 °C ± 2 °C and a relative humidity of 60% ± 5%. Standard food was available ad libitum for all animals. *Mcpt5-cre*^31^, *Tln1*^fl/fl^^32^, *Itgb1*^fl/fl^^33^, *Rosa26*^LSL:YFP^ (Jackson Laboratory, 006148)^34^, *Rosa26*^LSL:Tom^ (Jackson Laboratory, 007914)^35^, *Tg*(*Ubow*)^21^, *Tg*(*Kitl-ERT2cre*, *TdTomato*)^36^, *Kitl*^GFP^ (Jackson Laboratory, 017860)^37^, *Tyr*^c-2J^ (Jackson Laboratory, 000058)^38,39^, *Tg*(*Myh11-GFP*) (Jackson Laboratory, 007742)^40^, *Rag2*^tm1Fwa^^41^, *Tg*(*Lifeact-GFP*)^42^, *Itgb2*^tm2Bay^ (Jackson Laboratory, 003329)^43^, *Commd10*^Tg(Vav1-icre)^ (Jackson Laboratory, 008610)^44^ and *Itgb3*^fl/fl^ (Jackson Laboratory, 028232)^45^ mouse strains have been described elsewhere. *Mcpt5-cre Tln1*^fl/fl^ mice and crosses with fluorescent reporter lines (*Rosa26*^LSL:YFP^, *Tg*(*Ubow*)) were on a *Tyr*^c-2J/c-2J^ (C57BL/6J-Albino) background, as we initially planned intravital microscopy studies of ear skin in these mice. All other mouse strains were on a C57BL/6J background. For all genotypes, sex-matched female or male mice (aged 7 to 12 weeks) were used in the experiments.

### Culturing BMMCs with a connective tissue phenotype

To obtain MCs with connective tissue-type characteristics, we cultured BMMCs following previously published protocols^46,47^. BM was isolated by flushing tibiae and femora with cold PBS. Isolated BM cells were maintained at 37 °C and 5% CO_2_ in DMEM (4.5 g liter^–1^ glucose, Gibco) supplemented with 10% heat-inactivated fetal calf serum (FCS), 10 U ml^–1^ penicillin, 10 µg ml^–1^ streptomycin, 2 mM l-glutamine, 25 mM HEPES, 1 mM sodium pyruvate, 1× non-essential amino acids (Gibco), 50 µM 2-mercaptoethanol and interleukin-3 (IL-3; 5% supernatant of mouse IL-3-secreting WEHI-3 cells). To promote BMMC differentiation with connective tissue-type characteristics, 5% mouse SCF-containing supernatant and IL-4 (1 ng ml^–1^, PeproTech) were added to the medium^47^. IL-4 and SCF supplementation to the culture medium enhanced *Mcpt5* promoter activity and thus expression of Cre recombinase in BMMCs generated from *Mcpt5-cre* mouse strains. BMMCs were used for experiments after 5 to 10 weeks of cultivation. Full differentiation into mature MCs was confirmed by expression of lineage-specific c-KIT and FcεRI using flow cytometry.

### Peritoneal MCs

Mouse peritoneal MCs were isolated by peritoneal lavage and maintained at 37 °C and 5% CO_2_ in Opti-MEM including GlutaMAX (Life Technologies) supplemented with 10% heat-inactivated FCS, 10 U ml^–1^ penicillin, 10 µg ml^–1^ streptomycin (Gibco) and 5% mouse SCF-containing supernatant^48^. Experiments with peritoneal MCs (5 to 10 weeks of cultivation) were performed after confirming purity of mature MCs using flow cytometric analysis of lineage-specific c-KIT and FcεRI expression.

### Dermal MCs

Dermal MCs were isolated from the ear skin by separating ears into dorsal and ventral halves, mincing tissue and incubating for 75 min at 37 °C in RPMI supplemented with 50 U ml^–1^ penicillin, 50 µg ml^–1^ streptomycin and 0.4 mg ml^–1^ Liberase (Roche). The tissue digest was stopped by addition of an equal volume of RPMI supplemented with 10% heat-inactivated FCS. To obtain single-cell suspensions, digests were subjected to a gentleMACS dissociator (Miltenyi). After filtration of the suspension through a 70-µm filter (Corning), cells were cultivated for 5 weeks according to the protocol described for peritoneal MCs.

### IL-3, SCF and IgE production

IL-3 was produced by WEHI-3 cells^49^ (provided by R. Grosschedl, Max Planck Institute of Immunobiology and Epigenetics). SCF (KITLG) was produced by CHO transfectants (provided by G. Häcker, University of Freiburg). Anti-DNP IgE (clone SPE7) was produced by SPE-7 hybridoma NS1 cells^50^ (provided by M. Schmidt-Supprian, Technical University of Munich). Detailed information on culture conditions are available in Supplementary Note 1.

### Mouse embryonic fibroblast (MEF) culture and 3D fibroblast-derived FN matrices

Immortalized MEFs (provided by S. Minguet, University of Freiburg) were maintained as a confluent culture at 37 °C and 5% CO_2_ in DMEM (4.5 g liter^–1^ glucose; Gibco) supplemented with 10% heat-inactivated FCS, 2 mM l-glutamine, 50 U ml^–1^ penicillin, 50 µg ml^–1^ streptomycin and 50 μM β-mercaptoethanol. Stroma-derived 3D FN matrices were produced as previously described^51^. For matrix production, MEFs were seeded at 0.5 × 10^5^ cells per well in 8-well imaging chambers (Lab-Tek) for live-cell imaging or at 1.25 × 10^5^ cells per well in 24-well plates containing round coverslips (12 mm in diameter) for static immunofluorescence imaging. Cells were cultured for 9 d, and fluorescent labeling of matrices was achieved by adding 1 µg ml^–1^ rhodamine-coupled FN (Cytoskeleton) at days 3, 5 and 7, allowing incorporation of fluorescently labeled FN into the secreted matrices. For static immunofluorescence analysis, the extracted FN matrices were stained with anti-FN (Sigma) and fluorescently labeled anti-rabbit (Thermo Fisher).

### MC maturation and integrin expression

To confirm MC maturation and characterize integrin cell surface expression on MCs, flow cytometric analysis was performed; 10^6^ BMMCs per sample were used for staining and kept on ice. Antibodies and cells were diluted in PBS supplemented with 2% heat-inactivated FCS (FACS buffer). First, cells were blocked at 4 °C for 15 min using anti-mouse CD16/CD32 (1:250; BD Biosciences) before washing. Next, cells were stained for 30 min at 4 °C with primary antibodies and washed once with FACS buffer. If primary antibodies were unlabeled, cells were stained with fluorescently labeled secondary antibodies for 30 min. For flow cytometric measurements, MCs were resuspended in FACS buffer and analyzed using an LSRII or LSRFortessa flow cytometer operated with FACSDiva software (BD Biosciences). Final data analysis was done with FlowJo software (BD Bioscience). Antibodies and working dilutions are listed in the Reporting Summary. To measure integrin cell surface expression of endogenous skin MCs, flow cytometric analysis was performed on MCs isolated from mouse ear skin. Ears were separated into dorsal and ventral parts, finely minced on ice using sharp scissors and incubated with Opti-MEM (Gibco) containing 1 mg ml^–1^ Liberase DL (Roche, 05401160001) and 2 mg ml^–1^ DNase I (Roche, 10104159001) for 60 min at 37 °C while shaking at 1,500 r.p.m. in a thermomixer. Samples were collected on ice in a 50-ml reaction tube containing 10 ml of Opti-MEM. The suspension was filtered through a 30-µm cell strainer into a fresh 15-ml reaction tube and centrifuged for 5 min at 400*g* at room temperature, and the resulting cell pellet was washed once using 10 ml of Opti-MEM before the samples were centrifuged once more, and the supernatant was discarded. Antibody incubation was performed in 96-well round-bottom plates. Anti-CD16/32 blocking and staining were performed as for BMMCs. Antibodies and working dilutions are listed in the Reporting Summary. After staining with antibodies, the cells were washed once and taken up in FACS buffer supplemented with DAPI before flow cytometry.

### Calculation of TLN-1 depletion efficiency

To determine TLN-1 depletion efficiencies in BMMC and peritoneal MC cultures, intracellular flow cytometry and western blotting were performed. For intracellular detection of TLN protein expression by flow cytometry, BMMCs were fixed with 2% paraformaldehyde for 5 min on ice, permeabilized in 0.5% saponin and 0.5% heat-inactivated FCS in PBS (Perm buffer) for 10 min at room temperature and blocked with 2% mouse serum diluted in Perm buffer for 30 min. Cells were washed twice with Perm buffer. Unconjugated anti-pan-TLN (Sigma) was applied in 2% mouse serum-containing Perm buffer for 30 min on ice. Cells were washed twice with Perm buffer and incubated with Cy3-conjugated anti-mouse (Jackson ImmunoResearch) for 30 min on ice, followed by two subsequent washing steps with Perm buffer. Cells were resuspended in FACS buffer for flow cytometric analysis.

For western blotting, BMMCs and peritoneal MCs were lysed in freshly prepared RIPA buffer (50 mM Tris-HCl, 150 mM NaCl, 0.5% (vol/vol) NP-40, 1% (vol/vol) Triton X-100, 5 mM EGTA, 5 mM EDTA and 1× cOmplete protease inhibitor cocktail) for 15 min on ice. Proteins were applied to an 8–2% gradient polyacrylamide gel, resolved by SDS–PAGE (Bio-Rad) and transferred onto PVDF membranes (Millipore) via wet blot transfer. Nonspecific binding sites were blocked with 5% skim milk powder in TBS containing 0.1% (vol/vol) Tween 20. Primary antibodies to pan-TLN protein (Sigma) and actin (Sigma) were incubated overnight in TBS containing 0.1% Tween 20 and 2% bovine serum albumin (BSA; Sigma), followed by a 1-h incubation with horseradish peroxidase-conjugated secondary antibodies (Dako) at room temperature. After 5 min of incubation with Clarity Western ECL substrate (Bio-Rad), proteins were detected using a ChemiDoc Touch gel imaging system and Image Lab software (Bio-Rad).

### Adhesion assay

To measure MC adhesion to different ECM components and integrin ligands, an absorbance-based adhesion assay was performed. Microtiter plates (Greiner) were coated for 2 h at 37 °C with FN (10 µg ml^–1^; Sigma-Aldrich), recombinant human ICAM-1 (10 µg ml^–1^; R&D Systems), Matrigel (50 µg ml^–1^; Corning) or BSA as a control (250 µg ml^–1^) diluted in PBS. Plates were washed twice with PBS and blocked with 3% BSA in PBS for 30 min at 37 °C. BMMCs were incubated for 1 h at 37 °C in adhesion buffer (phenol red-free RPMI containing 10 mM HEPES, 0.25% BSA and 2 mM CaCl_2_) or in adhesion buffer supplemented with 1 µg ml^–1^ anti-DNP IgE. Cells were washed twice with PBS and seeded at 5 × 10^5^ cells per well in fresh adhesion buffer. Cells were allowed to settle for 10 min at 37 °C and 5% CO_2_. IgE-uncoated MCs were stimulated for 30 min with BSA (250 µg ml^–1^), anti-DNP IgE (1 µg ml^–1^) or SCF (100 ng ml^–1^). Anti-DNP IgE-sensitized MCs were stimulated with DNP–human serum albumin (DNP–HSA; 100 ng ml^–1^). After stimulation, plates were centrifuged upside down for 5 min at 60*g* to remove non-adherent cells. After washing once with prewarmed PBS, plates were centrifuged again upside down. Adherent cells were fixed for 10 min with 4% paraformaldehyde, followed by a washing step with PBS. Plates were centrifuged for 2 min at 420*g*, and adherent cells were stained by incubation with crystal violet (5 mg ml^–1^ in 2% ethanol) for 10 min at room temperature. Following two washing steps with tap water, plates were drained upside down, and 1% SDS (in water) was added. Plates were put on an orbital shaker to solubilize the crystal violet dye from lysed cells before absorbance was measured at 570 nm on a Synergy4 plate reader (Bio-Tek).

### MC spreading

To visualize adhesion-dependent MC spreading, live video microscopy was performed with a spinning-disk confocal microscope (Zeiss) equipped with a stage-top incubator (TokaiHit) to generate an ambient atmosphere of 37 °C and 5% CO_2_. Eight-well imaging chambers (Lab-Tek) were coated with 15 µg ml^–1^ FN overnight at 4 °C. After sensitizing BMMCs (non-fluorescent or Lifeact–GFP expressing) with 1 µg ml^–1^ anti-DNP IgE for 1 h at 37 °C, cells were washed twice in PBS and resuspended in imaging medium (phenol red-free DMEM supplemented with 10% heat-inactivated FCS, 10 U ml^–1^ penicillin, 10 µg ml^–1^ streptomycin, 5% IL-3-containing WEHI-3 supernatant and 1 mM CaCl_2_) and plated at 5 × 10^4^ cells per well. Microscopy was started immediately after addition of 60 ng ml^–1^ DNP–HSA. Differential interference contrast and confocal spinning-disk microscopy were used to generate time-lapse videos. Technical details on imaging are provided in Supplementary Note 1. MC adhesion to native FN matrices was investigated. Briefly, eight-well Lab-Tek imaging chambers containing rhodamine-labeled FN matrices were washed twice with PBS and filled with 30 µl of imaging medium. BMMCs were resuspended in imaging medium at a concentration of 10^6^ cells per ml, and 70 µl of cell suspension was added to the matrices for 1 h at 37 °C. To stimulate adhesion, 100 µl of SCF (final concentration of 100 ng ml^–1^) was added to the well. Technical details on imaging are provided in Supplementary Note 2. Raw imaging data were processed with Imaris (V9.5.1, Bitplane) using a Gaussian filter for noise reduction and displayed as 2D maximum intensity projections.

### MC migration in the under-agarose assay

MC migration in a confined environment was analyzed in an under-agarose assay. The standard protocol^52^ was modified to allow observation of (1) MC migration over a time course of 14 h and (2) MC actin flow dynamics. To investigate MC migration over time, custom-made imaging chambers (threaded tops of 50-ml centrifuge tubes (Cellstar) fixed in the center of Petri dishes 3.5 cm in diameter) were coated with 10 μg ml^–1^ FN in PBS overnight at 4 °C. Unbound FN was removed by washing the plates twice with PBS. UltraPure agarose (0.25 g; Invitrogen) was dissolved in 10 ml of double-distilled water by boiling and mixed with 30 ml of HBSS/DMEM solution (1:1 (vol/vol) HBSS/BMMC medium supplemented with 20% FCS, 5% IL-3-containing WEHI-3 supernatant and 5% SCF-containing CHO supernatant) to obtain a final gel concentration of 6.25 mg ml^–1^. To avoid denaturation of the FN layer, agarose gel was cast when the gel mixture was cooled down to a temperature of 40 °C. After polymerization, gels were equilibrated for 1 h at 37 °C and 5% CO_2_. BMMCs were sensitized with 1 µg ml^–1^ anti-DNP IgE for 1 h at 37 °C, followed by two washing steps with PBS. One microliter of the MC suspension (adjusted to 2 × 10^7^ cells per ml in BMMC medium) was injected under the agarose. The outer ring of the imaging chamber was filled with double-distilled water to avoid gel dehydration during the extended imaging time. Cells were incubated for 4 h at 37 °C before live-cell microscopy was started. Technical details on imaging are provided in Supplementary Note 2. For experiments with cytoskeletal inhibitors, 10^6^ anti-DNP IgE-sensitized BMMCs were incubated with 50 µl of inhibitor-containing BMMC medium for 1 h at 37 °C. Additionally, the agarose and agarose/HBSS solutions were supplemented with the corresponding inhibitor. The following inhibitors were used: cytochalasin D (1 µM; Merck) and Y-27632 (30 µM; Merck). DMSO and double-distilled water were used as a vehicle control for cytochalasin D and Y-27632, respectively. To investigate actin flow dynamics of MCs, we used total internal reflection fluorescence (TIRF) microscopy. Custom-made imaging chambers with glass-bottom slides (based on Petri dishes 3.5 cm in diameter with a 15-mm hole drilled into the center, which was covered with a glass coverslip 18 mm in diameter) were coated with 10 μg ml^–1^ FN diluted in PBS overnight at 4 °C. Control and *Tln1*^−/−^ BMMCs were generated from WT *Lifeact-GFP*^+/−^ or *Tln1*^ΔMC^ *Lifeact-GFP*^+/−^ mice, respectively. Agarose gels were cast and equilibrated for 1 h at 37 °C and 5% CO_2_. Before imaging, anti-DNP IgE-sensitized BMMCs were directly suspended in 60 ng ml^–1^ DNP–HSA solution at a concentration of 5 × 10^7^ cells per ml. One microliter of the cell suspension was injected under the agarose at five injection sites per well, and imaging was immediately started.

### MC migration in polydimethylsiloxane (PDMS) microchannels

Custom-ordered microchannels (9 µm and 10 µm in width and 10 µm in height; 4D Cell) were treated according to the manufacturer’s protocol. The channels were washed twice with PBS (Gibco) and coated with 100 µg ml^–1^ FN in PBS for 1 h at room temperature. Microchannels were washed three times each, first with PBS and then with BMMC medium. The medium was removed after the last wash step, and 10 µl of cell suspension (10^7^ cells per ml) was pipetted into each access port. After incubation at 37 °C and 5% CO_2_ for 30 min, 2 ml of BMMC medium was added and again incubated at 37 °C and 5% CO_2_ for 4 h before imaging was started. Technical details on imaging are provided in Supplementary Note 2.

### MC seeding of 3D Matrigel matrix

To assess MC migration and seeding in 3D matrices, BMMCs were kept in Matrigel for up to 72 h. Matrigel (Corning) was used according to the manufacturer’s protocol. BMMCs were sensitized with 1 µg ml^–1^ anti-DNP IgE for 1 h at 37 °C and 5% CO_2_. After washing twice with PBS, cells were suspended in BMMC culture medium at a concentration of 10^6^ cells per ml. Next, the BMMC suspension was mixed in a 1:1 ratio with BMMC medium containing 10% heat-inactivated FCS, 10% IL-3-containing WEHI-3 supernatant and 10% SCF-containing CHO supernatant on ice. Twenty-five microliters of this cell suspension was then mixed with 25 µl of Matrigel on ice to obtain a cell density of 2.5 × 10^4^ cells per 100 µl of Matrigel solution. For imaging, 10 μl of the gel mixture (2,500 cells in 50% (vol/vol) Matrigel per well) was loaded on an µ-slide angiogenesis (Ibidi, 81506) and kept at room temperature for 5 min and another 10 min at 37 °C and 5% CO_2_ to allow gel polymerization. Afterward, 50 μl of BMMC medium with 2× concentrated cytokines (10% IL-3-containing WEHI-3 and 10% SCF-containing CHO supernatant) was added on top of the gel to provide adequate culture conditions. The prepared samples were preincubated for 24 h at 37 °C and 5% CO_2_ and imaged afterward. Technical details on live-cell imaging are provided in Supplementary Note 2. For cluster growth analysis, Matrigel-embedded cells were incubated at 37 °C for 7 d before they were imaged. For experiments in the presence of cytoskeletal inhibitors, 10^6^ anti-DNP IgE-sensitized BMMCs were incubated in 50 µl of inhibitor-containing BMMC medium for 1 h at 37 °C. Additionally, inhibitors were added to both the Matrigel and BMMC medium that were loaded on top of the solidified Matrigel. The following inhibitors were used: blebbistatin (50 µM; Merck), cytochalasin D (1 µM; Merck) and Y-27632 (30 µM; Merck). DMSO was used as a vehicle control for blebbistatin and cytochalasin D, whereas double-distilled water was used for Y-27632.

### Ear skin whole-mount immunofluorescence analysis

Ears were cut at the base and subsequently split into ventral and dorsal halves. Ventral ear sheets were incubated in 1% paraformaldehyde for 6 to 8 h at 4 °C on a rocker. Ear slices were then transferred to wash and blocking buffer (PBS, 0.25% (vol/vol) Triton X-100 and 1% BSA) and incubated overnight by gently shaking at 4 °C. After fixation and permeabilization, immunofluorescence staining was performed. During all steps, ears were kept at 4 °C on a rocker. Each step was followed by three 15-min wash steps with wash buffer. Primary antibodies or directly fluorescent reagents were applied overnight at 4 °C. If required, this was followed by a 4- to 6-h incubation at 4 °C with fluorescent secondary antibodies. Tissue samples were mounted onto glass slides and covered by glass coverslips (the dermal side facing the coverslip) with Fluoromount-G (SouthernBiotech). Ear explants of Ubow mice were fixed for only 4 h in 1% paraformaldehyde at 4 °C to reduce quenching of the weak CFP fluorescence signal. Samples were otherwise treated according to the described protocol above. Antibodies and working dilutions are listed in the Reporting Summary. Anti-ACKR1 Alexa Fluor 488 (clone 6B7) was provided by Aude Thiriot and Ulrich von Andrian^53^ (Harvard Medical School). For amplification of Tomato signal in transgenic *Kitl-TdTomato* mice, anti-RFP (Rockland) and anti-rabbit Alexa Fluor 568 (Invitrogen) were used in some experiments. To amplify YFP signal in *R26*^LSL:YFP^-carrying mouse strains, anti-GFP Dylight 488 (Rockland) was used. Avidin was used to visualize dermal MCs, specifically avidin-FITC (1:2,000; BioLegend) and self-conjugated avidin-Alexa Fluor 647 (1:5,000; Thermo Fisher) using an antibody labeling kit (Thermo Fisher). Technical details on confocal fluorescence microscopy are provided in Supplementary Note 2.

### Immunofluorescence analysis of adhesion structures in MCs

Static immunofluorescence staining of adhesive structures in adherent MCs was performed on glass coverslips (12 mm in diameter). Coverslips were coated overnight at 4 °C with 15 µg ml^–1^ FN diluted in PBS. BMMCs were sensitized with 1 µg ml^–1^ anti-DNP IgE for 1.5 h at 37 °C and 5% CO_2_. Cells were washed twice with PBS and adjusted to 1.5 × 10^6^ cells per ml in assay medium (BMMC medium supplemented with 5% IL-3-containing WEHI-3 supernatant and 2 mM CaCl_2_). Unbound FN was aspirated from the coverslips, which were washed twice with PBS. Cells were plated in 400 µl of medium per well and allowed to settle for 10 min at 37 °C and 5% CO_2_. Next, 400 µl of stimuli solution (recombinant SCF (200 ng ml^–1^; Peprotech) and DNP–HSA (200 ng ml^–1^)) was added, and cells were incubated for 30 min at 37 °C and 5% CO_2_. Floating cells were aspirated, and adherent cells were fixed by adding prewarmed 1% paraformaldehyde (37 °C; diluted in PBS) for 15 min at 37 °C. Cells were washed twice with PBS before paraformaldehyde was quenched with 0.1 mM glycine for 5 min at room temperature. Afterward, the cells were permeabilized with 0.2% Triton X-100 in PBS for 5 min at room temperature and blocked for 30 min with 1% BSA. Primary antibodies were applied 1:200 in 1% BSA overnight at 4 °C. Coverslips were washed three times with 1% BSA for 5 min each. Cells were then stained with secondary antibodies for 90 min at room temperature. After three washing steps for 15 min each, coverslips were rinsed once with double-distilled water and mounted onto glass slides with antifade mounting medium. Antibodies and working dilutions are listed in the Reporting Summary. The actin cytoskeleton was visualized by using phalloidin conjugated to Alexa Fluor 488 (1:2,000; Thermo Fisher Scientific). Technical details on confocal fluorescence microscopy are provided in Supplementary Note 2. To evaluate the formation of adhesive structures in a more native setting, MCs were allowed to adhere to coverslips coated with FN matrices. BMMCs were treated, stimulated and fixed as described in the immunofluorescence protocol above. After permeabilization and blocking, primary antibodies were applied overnight at 4 °C followed by an incubation with secondary antibodies for 1 h at 4 °C. Antibodies and working dilutions are listed in the Reporting Summary.

### RNA FISH

To confirm results from RNA-seq experiments and analyze differential expression of proteins in ear skin tissue, RNA FISH by the third-generation in situ hybridization chain reaction (HCR) v3.0 protocol from Molecular Instruments was used according to the manufacturer’s protocol (Molecular Instruments Protocols). Ears from *Mcpt5-cre*^+/−^ *R26*^LSL:Tom^ mice were dissected following the previously described standard protocol and fixed with 4% paraformaldehyde overnight at 4 °C. In an RNase-free environment, ears were step-wise dehydrated in gradually increasing concentrations of methanol (25, 50, 75 and 100%) diluted in 0.1% Tween 20 in PBS (PBST) for 5 min each at room temperature and kept in 100% methanol overnight at −20 °C. Ears were rehydrated in gradually decreasing concentrations of methanol (100, 75, 50 and 25%) for 5 min each at room temperature before treatment with 10 µg ml^–1^ proteinase K solution (diluted in PBST) for 2 min at room temperature. Following a washing step, ears were fixed in 4% paraformaldeyde for 20 min at room temperature. For the detection stage, ears were incubated with preheated (37 °C) hybridization buffer for 5 min at room temperature and for another 30 min at 37 °C for prehybridization of the tissues. FISH probes (250 µl, 10 nM) were applied in hybridization buffer and incubated overnight at 37 °C. For the amplification stage, ears were incubated in 250 µl of amplification buffer containing 30 pmol of fluorescently labeled hairpin 1 and 2 and primary antibodies overnight at room temperature. Following three washing steps with 5× saline-sodium citrate buffer in distilled water and 0.1% Tween 20, ears were stained with secondary antibodies diluted in 5× saline-sodium citrate buffer in distilled water and 0.1% Tween 20 for 4 h at 4 °C. Following three washing steps in PBST at room temperature, ears were mounted onto glass slides using Fluoromount-G (SouthernBiotech). The following HCR probes and antibodies were used: *Tpbs2* (*Mcpt6*; ten probe pairs, Molecular Instruments), *Cma1* (ten probe pairs, Molecular Instruments), HCR amplifier Alexa Fluor 488 (Molecular Instruments), anti-nidogen (Thermo Fisher) and anti-rat Alexa Fluor 405 (Abcam). Technical details on imaging are provided in Supplementary Note 2.

### Cell isolation and FACS for scRNA-seq

All MCs used for scRNA-seq analysis were obtained from 7-week-old *Tln1*^ΔMC^ *R26*^LSL:YFP^ male mice (*n* = *3*) and age- and sex-matched control *Mcpt5-cre*^+/−^ *Tln1*^+/+^ *R26*^LSL:YFP^ littermate mice (*n* = *3*). Ears were separated into dorsal and ventral halves, finely minced and incubated for 75 min at 37 °C in RPMI supplemented with 5% penicillin–streptomycin and 0.4 mg ml^–1^ Liberase (Roche). Digestion was stopped by adding an equal volume of stopping solution consisting of RPMI supplemented with 10% FCS to the digestion mixture and keeping cells on ice. Single-cell suspensions were obtained using a gentleMACS dissociator (Miltenyi) and filtering the suspension through a 70-µm filter (Corning). For an unbiased selection of MCs and to avoid MC activation, we sorted for MC-specific YFP expression rather than using potentially activating lineage-specific markers. After excluding dead cells (DAPI) and doublets, MCs were identified as CD45^+^, Lin^−^ (CD3e^−^, CD19^−^, CD4^−^, CD8^−^, CD11c^−^ and NK1.1^−^) and YFP^+^ cells. Cells were sorted into 384-well plates using an Aria Fusion II (BD). Antibodies and working dilutions are listed in the Reporting Summary. All sorted cells expressed high transcript levels of the MC marker *Cpa3* (Extended Data Fig. 5b). For scRNA-seq analysis of VSMCs and pericytes, CD45^−^GFP^+^ cells were sorted from ear skin digests of *Myh11*^GFP/+^ mice.

### Single-cell RNA amplification and library preparation

scRNA-seq was performed according to the mCEL-Seq2 protocol^23,54^. Viable cells were sorted into 384-well plates containing 240 nl of primer mix and 1.2 μl of PCR encapsulation barrier, Vapour-Lock (Qiagen) or mineral oil (Sigma-Aldrich). Sorted plates were centrifuged at 2,200*g* for 10 min at 4 °C, snap-frozen in liquid nitrogen and stored at −80 °C until they were processed. To convert RNA into cDNA, 160 nl of reverse transcription reaction mix and 2.2 μl of second-strand reaction mix was used. cDNA from 96 cells was pooled together before cleanup and in vitro transcription, generating four libraries from one 384-well plate; 0.8 μl of AMPure/RNAClean XP beads (Beckman Coulter) per 1-μl sample was used during all purification steps including library cleanup. Libraries were sequenced on Illumina HiSeq 2500 and 3000 sequencing systems (paired-end multiplexing run, high-output mode) at a depth of ~150,000 to 200,000 reads per cell.

### Quantification of transcript abundance

Paired-end reads were aligned to the transcriptome using bwa (version 0.6.2-r126) with default parameters^55^. The transcriptome contained all gene models based on the mouse ENCODE VM9 release downloaded from the University of California Santa Cruz genome browser comprising 57,207 isoforms, with 57,114 isoforms mapping to fully annotated chromosomes (1–19, X, Y and M). All isoforms of the same gene were merged to a single gene locus. Subsequently, gene loci with >75% sequence overlap were merged. The right mate of each read pair was mapped to the ensemble of all gene loci and to the set of 92 ERCC spike-ins in the sense direction. Reads mapping to multiple loci were discarded. The left read contains the barcode information; the first six bases corresponded to the unique molecular identifier (UMI), followed by six bases representing the cell-specific barcode. The remainder of the left read contains a poly(T) stretch. The left read was not used for quantification. For each cell barcode, the number of UMIs per transcript was counted and aggregated across all transcripts derived from the same gene locus. The number of observed UMIs was converted into transcript counts using binomial statistics^56^.

### scRNA-seq data analysis

Clustering and visualization were performed using the RaceID3 (v0.2.3) algorithm^23^. Cells expressing >2% of Kcnq1ot1, a potential marker for low‐quality cells^57^, were not considered for the analysis. For normalization, the total transcript counts in each cell were normalized to 1 and multiplied by the minimum total transcript count across all cells that passed the quality control threshold of >1,000 transcripts per cell (Poisson-corrected UMIs^57^). For the MC data, 1,895 cells passed the quality control threshold. For the smooth muscle cell data, 330 cells passed the quality control threshold. RaceID3 was run with the following parameters: mintotal = 1,000, minexpr = 5 and minnumber = 5. Mitochondrial and ribosomal genes as well as genes with Gm-identifiers were used as input for CGenes.

### Differential gene expression analysis

Differential gene expression analysis between cells and clusters was performed using the diffexpnb function from the RaceID3 package. First, negative binomial distributions reflecting the gene expression variability within each subgroup were inferred on the basis of the background model for the expected transcript count variability computed by RaceID3. Using these distributions, a *P* value for the observed difference in transcript counts between the two subgroups was calculated and corrected for multiple testing using the Benjamini–Hochberg method, as previously described^58^. UMAP representation was done as previously published^59^.

### Analysis of MC morphologies, spreading and migration

Detailed information on the analysis of MC spreading on 2D surfaces, MC migration and actin dynamics in confined spaces, MC cluster formation in Matrigel and MC morphologies in tissues is provided in Supplementary Note 3.

### Calculation of MC density in tissues

Detailed information is provided in Supplementary Note 3.

### Analysis of MC coverage and proximity to vessels

Detailed information on the analysis of MC coverage and proximity to vessels, proximity of MCPT6^high^ MCs to arterioles and MC arteriolar coverage is provided in Supplementary Note 4.

### Analysis of Ubow mice

To quantify MC cluster formation in Ubow mice, ear skin whole mounts were imaged using a LSM780 (Zeiss) confocal microscope equipped with a Plan-Apochromat ×20 M27 objective (Zeiss). *Z* stacks of 25 to 42 µm with a 2-µm step size and multiple tiles were acquired using ZEN black software, and 2 × 2 tiled images (1.2 × 1.2 mm; covering a minimum of 600 MCs) were stitched using ZEN blue software. Statistical analysis and quantification of spatial MC distribution were performed using ClusterQuant2D software^21,60^. Detailed information of the analysis is provided in Supplementary Note 5.

### Quantification of RNA FISH and MCPT6 expression in MCs

Detailed information on the quantification of RNA FISH and MCPT6 expression in situ is provided in Supplementary Note 6.

### Statistics and reproducibility

Analyses were performed using Prism software (GraphPad Software, version 9.3.1). No statistical methods were used to predetermine sample size and were determined based on previous publications^61,62^, prior experience or pilot experiments. Sample sizes for biological replicates in cell culture experiments (that is, cultures generated from different individual mice) aimed for a minimum of mouse donors to reduce the number of laboratory animals. Data exclusion methods were not applied for most experiments. Exceptions are listed in the Reporting Summary. If not indicated otherwise, comparisons for two groups were evaluated using a two-sided unpaired *t*-test after confirming that samples fulfilled the criteria of normal distribution and equal variance. The D’Agostino and Pearson normality test was used for formal testing of group sizes over ten. For testing of group sizes below ten, the Shapiro–Wilcoxon normality test was performed. For non-Gaussian-distributed data, non-parametric two-sided Mann–Whitney *U*-tests were used for comparisons of two groups. Experimental groups were defined by inhibitor treatment or by the genotype. MCs and MC clusters were randomly chosen for tracking, cell size analysis or cluster size determination, respectively. Experimentalists were blinded regarding the genotype of mice or BMMC cultures. Mouse numbers were used as identifiers. Stars indicate significance (*P ≤ 0.05, **P ≤ 0.01 and ***P ≤ 0.001). Non-significant differences are indicated as NS (*P* > 0.05). For further details on statistical tests, please see Supplementary Table 1. If not indicated in the figure legends, representative micrographs are based on independent experimental repeats with similar results: *n* = 3 mice (Figs. 1a,b and 5c–i), *n* = 5 cells from one of two independent experiments (Fig. 1c,d), *n* = 10 cells from one of three independent experiments (Fig. 1e), *n* = 8 mice per each genotype (Fig. 3b), *n* = 3 independent experiments (Fig. 3h,i) and *n* = 5 mice (Fig. 5j).

### Reporting summary

Further information on research design is available in the Nature Portfolio Reporting Summary linked to this article.

## Online content

Any methods, additional references, Nature Portfolio reporting summaries, source data, extended data, supplementary information, acknowledgements, peer review information; details of author contributions and competing interests; and statements of data and code availability are available at 10.1038/s41590-023-01493-2.

## Supplementary information

Supplementary Information Supplementary Notes 1–6 and Table 1.

Reporting Summary

Supplementary Video 1 Integrin-dependent MC spreading on FN-coated 2D surfaces. MCs were cultivated from *Tln1*^ΔMC^ *Lifeact-GFP*^+/−^ and WT *Lifeact-GFP*^+/−^ mice, coated with anti-DNP IgE and applied to FN-coated 2D surfaces. DNP–HSA was added to induce FcεRI-mediated MC spreading. Spinning-disk confocal microscopy of cellular actin revealed that WT MCs adhered, spread and started to migrate, whereas *Tln1*^−/−^ MCs did not adhere and spread. This video relates to Fig. 1g.

Supplementary Video 2 Integrin-dependent MC motility on fibroblast-derived FN matrix. MCs were cultivated from *Tln1*^ΔMC^ *Lifeact-GFP*^+/−^ and WT *Lifeact-GFP*^+/−^ mice and applied to rhodamine-labeled FN matrices in the presence of SCF. Video sequences are displayed on top, and static side views are displayed on the bottom. Spinning-disk confocal microscopy revealed that WT MCs integrate, interact and move into the matrix. By contrast, *Tln1*^−/−^ MCs fail to invade the matrix and float on top of the FN layer. This video relates to Extended Data Fig. 3e.

Supplementary Video 3 Integrin-dependent MC migration in a confined space (under-agarose assay). MCs were cultivated from *Tln1*^ΔMC^ and littermate control mice and applied to an under-agarose assay. MC movement occurred in the confined space between the agarose layer and the FN-coated cell culture plastic underneath. Phase contrast brightfield microscopy over 14 h revealed random movement and lamellipodia formation of WT MCs. *Tln1*^−/−^ MCs hardly moved and remained largely immotile. This video relates to Extended Data Figs. 3f,i,j.

Supplementary Video 4 Lack of high-affinity integrins increases the retrograde actin flow in MCs. Lifeact–GFP-expressing MCs were confined between an agarose layer and an FN-coated cell culture plastic, and lamellopodial actin dynamics were recorded. TIRF microscopy revealed protrusive lamellipodia movement without measurable retrograde actin flow in WT MCs, whereas *Tln1*^−/−^ MCs displayed a clear increase in retrograde actin flow. This video relates to Extended Data Fig. 3k.

Supplementary Video 5 Integrin-dependent MC migration in a confined space (PDMS microchannels). MCs were cultivated from *Tln1*^ΔMC^ and littermate control mice and applied to the confined space of 10 µm × 10 µm PDMS microchannels. A representative selection of ten channels per genotype is displayed. Phase contrast brightfield microscopy over 100 min revealed clear movement of WT MCs in these confined spaces. By contrast, *Tln1*^−/−^ MCs, which had managed to invade the microchannel, did not show active crawling behavior during this time frame. This video relates to Fig. 1i–l.

Supplementary Video 6 MC seeding in 3D matrix space requires integrin-dependent migration. MCs were cultivated from *Tln1*^ΔMC^ and littermate control mice, applied to 3D Matrigel in the presence of SCF and observed over 60 h by phase contrast brightfield microscopy. The movement of WT MCs is displayed first and includes fate tracking of two-cell clones and corresponding descendant cells. This video shows how proliferation and integrin-dependent migration shape the formation of a cellular network with heterogeneous cell distribution. The second part of the video shows how the majority of *Tln1*^−/−^ MCs are unable to move away from each other after cell division, which results in the growth of MC clusters. This video relates to Fig. 3g–i.

Supplementary Video 7 Rare example of an individual MC escaping a *Tln1*^−/−^ proliferation cluster and forming a new seed for another cluster. *Tln1*^−/−^ MCs were applied to 3D Matrigel in the presence of SCF and observed over 9 h by phase contrast brightfield microscopy. The majority of *Tln1*^−/−^ MCs are proliferating, but these cells are unable to move away from each other after cell division, which results in the formation of MC clusters. This video highlights a rare example of an escapee MC, which moves away from the cell cluster and forms a new seed for a new cell cluster. This video relates to Extended Data Fig. 6g.

Supplementary Video 8 Positioning of MCs in the SCF-rich periarteriolar niche with direct contact to SCF-expressing periarteriolar fibroblasts. This video shows an animated view of SCF expression in the arteriolar tissue niche. Ear skin explants of transgenic *Kitl-TdTomato* mice were fixed and counterstained for avidin (MC, cyan), α-smooth muscle actin (VSMCs) and collagen IV (basement membrane). Confocal fluorescence microscopy and 3D reconstruction of the confocal image stacks reveal a direct contact of periarteriolar MCs with SCF-expressing fibroblasts, which form an outer cellular layer in the periarteriolar space. This video relates to Fig. 5g,h.

## Data Availability

scRNA-seq data have been deposited in Gene Expression Omnibus under the accession code GSE205412. Genome annotation ENCODE VM9 (https://hgdownload.soe.ucsc.edu/gbdb/mm10/) was used for mapping scRNA-seq data. Source data are provided with this paper. All other data supporting the findings of this study are available within the article and supplementary materials.

## Source data

Source Data Fig. 1 Statistical source data.
Source Data Fig. 2 Statistical source data.
Source Data Fig. 3 Statistical source data.
Source Data Fig. 4 Statistical source data.
Source Data Fig. 5 Statistical source data.
Source Data Extended Data Fig. 2 Unprocessed western blots.
Source Data Extended Data Fig. 2 Statistical source data.
Source Data Extended Data Fig. 3 Statistical source data.
Source Data Extended Data Fig. 4 Statistical source data.
Source Data Extended Data Fig. 5 Statistical source data.
Source Data Extended Data Fig. 6 Statistical source data.

## Code availability

ClusterQuant2D code is provided at https://zenodo.org/record/7684553. Kymograph code is provided at https://github.com/KMGlaser/Bambach-et-al.git.
