# Supplementary material for: Slow integrin-dependent migration organizes networks of tissue-resident mast cells
Source: Nat Immunol. 2023 Apr 20;24(6):915–24. doi: 10.1038/s41590-023-01493-2 (PMC10232366; doi:10.1038/s41590-023-01493-2)
Supplement: Supplementary file 1 — Supplementary Notes 1–6 and Table 1. [file 41590_2023_1493_MOESM1_ESM.pdf]

nature immunology

Letter https://doi.org/10.1038/s41590-023-01493-2

Slow integrin-dependent migration organizes networks of tissue-resident mast cells

In the format provided by the authors and unedited

Supplementary Information:

Supplementary Notes 1–6

Supplementary Table 1

Supplementary Note 1

Details on IL3, SCF and IgE production

IL3 production by WEHI-3 cell line

WEHI-3 cells were cultured in DMEM (4.5 g/L glucose, 110 mg/L pyruvate; Gibco) supplemented with 10% heat-inactivated FCS, 10 U/ml penicillin, 10 µg/ml streptomycin, 2 mM L-glutamine and 50 µM 2-mercaptoethanol. Cells were kept at a concentration of 10^5 cells/ml and medium was exchanged twice a week. For conditioned media production, cells were incubated for 3 to 4 days until the cell concentration reached 1×10^6 cells/ml. Supernatant was collected, centrifuged for 10 min at 350 × g, filtered through a 0.45 µm filter (Nalgene) and kept at −20°C for long-term storage.

SCF production by CHO transfectants

CHO cells were kept at 37°C and 5% CO_2 in OptiMEM™ with Glutamax™ (Life Technologies) supplemented with 10% heat-inactivated endotoxin-low FCS, 10 U/ml penicillin, 10 µg/ml streptomycin and 30 µM 2-mercaptoethanol. Cells were passaged twice a week and maintained as a confluent culture. For SCF production, cells were seeded at a concentration of 7×10^5 cells per 15 cm cell culture plate (Greiner). When CHO cells reached 70% confluency, supernatant was collected and replaced by fresh medium. CHO supernatant harvest was repeated three to four consecutive days. The supernatant was centrifuged for 5 min at 500 × g, filtered through a 0.2-µm filter and kept at −20 °C.

IgE production

Anti-dinitrophenyl (DNP) IgE antibody (clone SPE7) was produced by SPE-7 hybridoma NS1 cells, which were cultured to a density of 0.5–1 million cells/ml at 37°C and 5% CO_2 in RPMI supplemented with 10 % FCS, 50 µM 2-mercaptoethanol, and 10 U/ml penicillin. The medium was changed to RPMI supplemented with 1 % FCS, 50 µM 2-mercaptoethanol and 10 U/ml penicillin for two days. Then, the supernatant was collected by centrifugation and sterile filtered. The supernatant was subsequently concentrated using a Sartocon® slice 200 cassette (Sartorius) by the Protein Production Core Facility of the Max Planck Institute of Biochemistry (Martinsried, Germany).

Supplementary Note 2

Technical details on microscopy and imaging

Mast cell spreading

For differential interference contrast (DIC) time-lapse videos of mast cell spreading to plate-bound fibronectin, a Plan-Apochromat 40×/ 1.3 Oil DIC Vis-IR M27 objective (Zeiss) was used and images were acquired every 20 s for 1 h. Multiple tiles were recorded to capture a sufficient number of cells. Image tiles were stitched with ZEN blue software. For confocal spinning-disk microscopy time-lapse videos, a Plan-Apochromat 63×/ 1.4 Oil DIC M27 objective (Zeiss) was used and images acquired every 10 s over 8 min. For the 3D reconstruction of MC adhesion to plate-bound fibronectin molecules, 30 to 50 µm *z*-stacks of individual Lifeact-GFP expressing MCs were acquired with 0.5 µm *z*-step size. To study mast cell adhesion to native fibronectin matrices, time-lapse videos were recorded using a Plan-Apochromat 40×/1.3 Oil DIC Vis-IR M27 objective (Zeiss) with image acquisition every 30 s over 30 min. 20 µm *z*-stacks with 0.5 µm z-step size were acquired. For confocal spinning-disk microscopy a Cell Observer SD system (Zeiss) consisting of a CSU-X1 confocal scanner unit (Yokogawa) mounted on an AxioObserver Z1 inverted microscope stand and equipped with Evolve back-illuminated EM-CCD camera (Teledyne Photometrics) was used. Depending on the used fluorochromes, images were acquired using laser-line excitation by 488 nm or 561 nm solid-state lasers. For DIC microscopy, a prime BSI scientific CMOS (sCMOS) camera was used.

Mast cell migration in confined spaces of the under-agarose assay

Mast cell migration over time: To analyze MC migration, an Axiovert 200 microscope (Zeiss) equipped with a heating chamber set to 37°C and a CO_2-controller (Zeiss) adjusted to 5% CO_2 was used. Time-lapse videos were recorded using a Plan Apochromat 10×/0.25 Ph1 objective (Zeiss). Images were acquired every 10 min for 14 h.

Mast cell actin flow dynamics: TIRF microscopy was performed using an Eclipse Ti-E inverted microscope (Nikon) equipped with an A1R confocal laser scanning system (Nikon). Images were acquired every 250 ms for 5 to 10min using a Plan Apo TIRF 60× Oil DIC H N2 objective (Zeiss).

Mast cell migration in confined spaces of PDMS microchannels

Brightfield live cell imaging was performed with an Axiovert 200 microscope (Zeiss) equipped with a heating chamber set to 37°C and a CO_2-controller (Zeiss) set to 5% CO_2. Cells were imaged in a 25- to 30-µm *z*-stack with 2-µm stepping every 10 min for 36 h using a Plan Apochromat 10×/0.25 Ph1 objective (Zeiss). For high-magnification imaging of Lifeact GFP

cells in microchannels, a confocal spinning-disk microscope (Zeiss) equipped with a stage top incubator (TokaiHit) was used. Cells were imaged in a 25- to 30-µm *z*-stack with 0.9 µm stepping using a Plan Apochromat 40×/1.3 Oil M27 objective (Zeiss) every minute for 14 min.

Mast cell seeding of 3D matrigel matrix

Live cell imaging was performed using an Axiovert 200 phase-contrast microscope (Zeiss) equipped with a heating chamber and a CO_2-controller (Zeiss) to generate an ambient atmosphere of 37°C and 5% CO_2 as described before. Time-lapse videos were recorded up to three days with image acquisition every 10 min and multiple positions to record sufficient cluster formation events using a Plan Apochromat 10×/0.25 Ph1 objective (Zeiss). To correct for a possible optical drift, *z*-stacks with 10 µm step size were acquired. Medium was exchanged every two days.

Ear skin whole mount immunofluorescence analysis

Either *z*-stacks or single focal planes were acquired (indicated in the respective analysis section and/or figure legend). Standard ear whole mount microscopy was performed using an inverted LSM 780 microscope (Zeiss). 3 × 3 tile images were acquired covering 16 to 30 µm of *z*-stack with a step size of 1 or 2 µm using a Plan Apochromat 20×/ 0.8 air objective (Zeiss). Tiled images were stitched during post-processing with ZEN blue software. Images were acquired using excitation through solid-state lasers (UV405; Argon488, DPSS561, HeNe633). The internal photomultiplier tubes and GaAsP detector of the confocal system were used for collecting the emitted fluorescence light.

Immunofluorescence analysis of adhesion structures in mast cells

Imaging was performed at a LSM 780 (Zeiss) confocal microscope using a Plan Apochromat 63×/ 1.4 Oil objective (Zeiss). *Z*-stacks were acquired in a range of 7 to 10µm with a step size of 0.51 µm. For deconvolution, cells were acquired starting above and ending under the object of interest, where relevant structures were slightly out of focus with a voxel size of 0.05 µm/voxel in *xy* and 0.51 µm/voxel in *z*. Huygens (SVI) software was used for deconvolution.

RNA fluorescence in situ hybridization (RNA-FISH)

Imaging was performed at a LSM 880 (Zeiss) equipped with an Airyscan detector and a Plan Apochromat 20×/0.8 M27 objective (Zeiss). Z-stacks with 0.4 µm step size and 2 x 2 tiles (0.45 x 0.45 mm) were acquired. A 32-channel GaAsP PTM detector of the confocal system was used for collecting the emitted fluorescence light. The FISH signal was detected

separately by the integrated Airyscan detector (optimal resolution mode) to improve resolution and signal-to-noise ratio and to avoid channel bleed-through.

Supplementary Note 3

Detailed information on the analysis of mast cell morphologies, spreading and migration

Analysis of mast cell spreading on 2D surfaces

To evaluate MC spreading (PMCs, BMMCs and ear skin MCs), snapshots of indicated timepoints were analyzed with ImageJ (V2.1.0/1.53). The areas of all cells in a region of interest were manually measured. Mean and standard deviation were calculated using GraphPad Prism.

Analysis of mast cell migration and actin dynamics in confined spaces

Live cell tracking of MCs in under-agarose assays and PDMS microchannels was performed using the manual tracking option of Imaris 9.1.2 (Bitplane). Cells were randomly chosen and tracked manually through all time frames. The average speed was calculated by dividing the total track length through the total time of imaging in min. Actin dynamics were measured and analyzed via total interference reflection (TIRF) microscopy. Background was subtracted from raw TIRF images using ImageJ (rolling ball algorithm, rolling ball radius 25 pixels) and a FFT bandpass filter was applied (3−40 pixels, no stripe suppression, 5% tolerance of direction). Kymographs were analyzed from filtered image sequences using a custom written Matlab program that allows for assembling of kymographs from arbitrary rectangular selections from leading edges of individual cells (code provided at https://github.com/KMGlaser/Bambach-et-al.git). Actin flow speeds were measured from kymographs by determining the slope of hand-drawn lines that follow prominent actin peaks over time.

Analysis of mast cell cluster formation in matrigel

To analyze differences in the cluster formation between *Tln1*$^{-/-}$ and control MCs, the areas of 20 randomly chosen MC clusters after seven days of growth in Matrigel™ were measured using ImageJ (V2.1.0/1.53). To quantify MC cluster growth over three days, images were taken every 20 min. Areas of 20 randomly chosen clusters were manually measured using ImageJ.

Mast cell morphologies in tissues

To assess MC morphology, YFP-reporter expression in *Tln1*$^{\Delta MC}$ *Rosa26*$^{LSL:YFP}$ mice (n = 3) and control littermates (n = 4) was used. For MC morphology analysis of *Itgb1*$^{\Delta MC}$ mice (n = *3*) and control littermates (n = *3*), avidin staining was used to determine the cell shape. Ear whole mounts were acquired with an inverted LSM 780 microscope (Zeiss). A Plan-Apochromat 40×/1.4 Oil DIC M27 objective (Zeiss) was used and 15 to 30 µm *z*-stacks with 1

µm step size were acquired with a zoom of 1.2. For 3D reconstruction and morphometric analysis Imaris (V9.1.2, Bitplane) was used. Morphology of MCs was evaluated by measuring the cellular areas manually in ImageJ (V2.1.0/1.53) and using the shape descriptor “circularity” = 4π × (area/perimeter^2).

Determination of mast cell density in tissues

To determine MC density in ear skin tissue, ear skin whole mounts of *Tln1*$^{\Delta MC}$ mice (n=11) and WT mice (n=11) with an age between 7 to 10 weeks were imaged using a LSM 780 microscope (Zeiss) equipped with a Plan-Apochromat 20× M27 objective (Zeiss). 18 to 25 µm thick *z*-stacks with 1 µm step size, 2 × 2 tiled images with 10% overlap and zoom of 1.0 were acquired with ZEN black software and stitched during post-processing with ZEN blue software. Four imaging fields of view, displayed as maximum intensity projection, were analyzed per mouse using Imaris (V9.1.2, Bitplane). MCs were identified using the spot function based on the avidin signal. MC density was displayed per analyzed tissue area in in mm^2.

Supplementary Note 4

Detailed information on the analysis of mast cell coverage and proximity to vessels

Mast cell coverage and proximity to vessels

To evaluate MC proximity to different anatomical structures and to measure the coverage of arterioles by MCs, images of ear skin whole mounts were acquired using a LSM 780 microscope (Zeiss) equipped with a Plan-Apochromat 20× M27 objective (Zeiss). 18 to 25 µm thick *z*-stacks with 1 µm step size were imaged. To capture sufficient numbers of cells, 2 × 2 tile images with 10% overlap were acquired with ZEN Black software and stitched during post-processing with ZEN blue software. Four imaging field of views (including ACTA2-positive structures) per mouse were analyzed. Mice were between 7 to 10 weeks of age. Periarteriolar alignment analysis was performed using Imaris (V9.1.2, Bitplane). After applying background subtraction and a Gaussian filter to all channels, the surface for endomucin (EMCN)-positive structures was created. Arteriolar surfaces were created after generating a new channel by discarding signal from ACTA2 voxel within endomucin surfaces. For venules, another channel was created by excluding signal from ACTA2 voxel outside endomucin surfaces. To retrieve data on MC proximity to ACTA2-positve arterioles and ACKR1-positive venules, the arteriolar surface was created after generating a new channel by discarding signal from ACTA2 voxel within a previously generated ACKR1 surface. For further details on this image analysis, see also Extended Data Fig. 4c, d. Tissue-resident MCs were identified using the spot function based on the avidin signal and an estimated diameter of 11 µm. MC alignment to arterioles, venules or capillaries was determined using the Xtension function “find spots close to surface” and setting the threshold to 5 µm.

Proximity of MCPT6^{high} mast cells to arterioles

To evaluate proximity relationships of MCPT6^{high} expressing MCs to arterioles, surfaces for the pan-MC marker avidin and ACTA2-positive structures were created. The mean intensity of the MCPT6 signal within MCs was used to create MCPT6^{high} and MCPT6^{low} channels. To calculate the proximity of MCPT6^{high} and MCPT6^{low} MCs to arterioles we used the Imaris Xtension tool “find spots close to surface”. Only MCs with a distance between 0-15 µm to arterioles were defined as closely located. Cells at ACTA2-positive structures without characteristic arteriolar morphology (capillaries) were manually excluded from the analysis. For further details on this imaging analysis, see also Extended Data Fig. 8c, d.

Mast cell arteriolar coverage

Using Fiji, the length of arteriolar walls and the length of MCs in direct contact to arterioles were manually measured in 2-dimensional maximum-intensity projections of acquired *z*-stacks. Coverage of arterioles by MCs was defined as follows: (Sum (length of arteriolar MCs)/Sum (length of arteriolar wall)) x 100.

Supplementary Note 5

Detailed information on the analysis of Ubow mice

Analysis of Ubow mice

YFP- and CFP-positive MCs were defined as two distinct classes using the Region-of-interest plugin (ROI manager) of the ClusterQuant2D software. Respective YFP- and CFP-positive MCs were then manually annotated to generate information on cell center coordinates. Counterstaining with avidin was used to distinguish MCs from potential auto-fluorescence. Next, 2-dimensional Voronoi meshes were generated based on the cell center coordinates. These diagrams show neighborhood relationships between cells of the same color and allow assessment of spatial distribution of cells in means of diffuse cell spreading or cluster formation (and cluster number/size). These data were used for analysis. For further details on this image analysis, see also Extended Data Fig. 6b. Average of cell numbers per cluster (cluster size) was used for analysis of the clustering index (Extended Data Fig. 6c).

Supplementary Note 6

Detailed information on the quantification of RNA-FISH and MCPT6 expression *in situ.*

Quantification of RNA-FISH and MCPT6 expression in mast cells

Analysis was performed on stitched tile images comprising 25 to 75 MCs using Imaris (V9.5.1, Bitplane). First, autofluorescent erythrocytes within the vasculature (using nidogen signal as mask) were excluded. Then, a surface for avidin-positive MCs was created and FISH signal was measured within this mask. Arteriolar aligned and interstitial MCs were manually discriminated. For each class a new surface was created and the according FISH signal was detected using the spot function. Thresholding filters were manually adjusted to detect all FISH signals. The Xtension "split into surfaces" was used to distinguish between FISH spots of individual cells. For quantification, the number of spots (FISH signal) within a cell was divided by the volume of an individual cell. To evaluate MCPT6 expression in arteriolar and interstitial MCs, images were acquired with a Plan Apochromat 25×/0.8 objective (Zeiss) at a LSM 780 (Zeiss). Z stacks of 25 to 35 µm with 2 µm step size were acquired followed by the stitching of the 2 × 2 filed images using ZEN blue software. After creating a surface for avidin-positive MCs, cells were manually distinguished into arteriolar and interstitial MCs. MCs aligning to ACTA2-positive structures without characteristic arteriolar anatomy were excluded from the analysis. The mean fluorescence intensity per cell of arteriolar and interstitial MCs was quantified.

Supplementary Table 1

Details on statistical tests

Figure	Statistical test		Summary
**1h**	Mann-Whitney (two-sided)	*U*=908, *n*=189,92 , *P*<0.0001	***
**1l**<br>9 µm<br>10 µm	 Mann-Whitney (two-sided)<br>Mann-Whitney (two-sided)	 *U*=7, *n*=22,16 , *P*<0.0001<br>*U*=3, *n*=19,20 , *P*<0.0001	 ***<br>***
**2c**<br>Art.<br>Ven.<br>Cap.	 unpaired *t* test (two-sided)<br>Mann-Whitney (two-sided)<br>Mann-Whitney (two-sided)	 *t*=5.570, *df*=46, *P*<0.0001, n(WT)=24, n(KO)=24<br>*U=252*, *P*=0.4645, n(WT)=24, n(KO)=24<br>*U=218.5, n=24,24 , P=0.1546*	 ***<br>ns<br>ns
**2d**	unpaired *t* test (two-sided)	*t*=17.10, *df*=38, *P*<0.0001, n(WT)=20, n(KO)=20	***
**2f**	Mann-Whitney (two-sided)	*U*=3.5, *n*=31,31 , *P*<0.0001	***
**3e**	Mann-Whitney (two-sided)	*U*=33, *n*=23,20 , *P*<0.0001	***
**3g**	Mann-Whitney (two-sided)	*U*=0, *P*<0.0001, n(WT)=20, n(KO)=20	***
**4h**	unpaired *t* test (two-sided)	*t*=5.072, *df*=53, *P*<0.0001, n(arteriolar MCs)=17, n(interstitial MCs)=38	***
**4i**	Mann-Whitney (two-sided)	*U*=109, *P*=0.0009, n(arteriolar MCs)=18, n(interstitial MCs)=28	***
**4l**	unpaired *t* test (two-sided)	*t*=5.718, *df*=49, *P*<0.0001, n(arteriolar MCs)=30, n(interstitial MCs)=21	***
**5k**	unpaired *t* test (two-sided)	*t*=5.913, *df*=25, *P*<0.0001, n(WT)=15, n(KO)=12	***
**Extended Data Figure**			
**2f**	unpaired *t test (two-sided)*<br>unpaired *t test (two-sided)*	*t=0.08533,df=4,P*=0.9361, n(WT)=3, n(KO)=3<br>*t=1.446,df=4,P*=0.221707, n(WT)=3, n(KO)=3	ns<br>ns
**3d**<br>DMC<br>PMC	 Mann-Whitney (two-sided)<br>unpaired *t* test (two-sided)	 *U*=7112, *P*=0.0001, n(WT)=137, n(KO)=141<br>*t*=10.23, *df*=14, *P*<0.0001, n(WT)=8, n(KO)=8	 ***<br>***
**3g**	Mann-Whitney (two-sided)	*U=0*, *P*<0.0001, n(Vehicle)=20, n(CytD)=20	***
**3h**	Mann-Whitney (two-sided)	*U*=23, *P*<0.0001, n(Vehicle)=20, n(Y-27632)=20	***
**3j**	Mann-Whitney (two-sided)	*U*=0, *n*=25, 25 *P*<0.0001	***
**3k**	unpaired *t* test (two-sided)	*t*=5.458, *df*=9, *n*=7, 4, *P*=0.0004	***
**4b**	unpaired *t* test (two-sided)	*t*=0.5290, *df*=20, *P*=0.6026, n(WT)=11, n(KO)=11	ns
**4d**<br>Art.<br>Ven.	 unpaired *t* test (two-sided)<br>unpaired *t* test (two-sided)	 *t*=5.368, *df*=22, *P*<0.0001, n(WT)=12, n(KO)=12<br>*t*=0.5290, *df*=22, *P*=0.4370, n(WT)=12, n(KO)=12	 ***<br>ns
**5c**	unpaired *t* test (two-sided)	*t*=7.461, *df*=38, *P*<0.0001, n(WT)=20, n(KO)=20	***
**5d**	Mann-Whitney (two-sided)	*U*=0, *P*<0.0001, n(WT)=20, n(KO)=20	***

**5e**	Mann-Whitney (two-sided)	*U*=514.5, *n*=59, 91, *P*<0.0001	***
**6c**<br>CFP<br>YFP	 unpaired *t* test (two-sided)<br>unpaired *t* test (two-sided)	 *t*=9, *df*=16, *P*<0.0001, n(WT)=10, n(KO)=8<br>*t*=15.71, *df*=16, *P*<0.0001, n(WT)=10, n(KO)=8	 ***<br>***
**6e**	unpaired *t* test (two-sided)	*t*=14.23, *df*=38, *P*<0.0001, n(Vehicle)=20, n(CytD)=20	***
**6f**<br>left<br>right	 unpaired *t* test (two-sided)<br>unpaired *t* test (two-sided)	 *t*=3.332, *df*=38, *P*=0.0019, n(Vehicle)=20, n(Y-27632)=20<br>*t*=3.010, *df*=38, *P*=0.0046, n(Vehicle)=20, n(Bleb)=20	 **<br>**
