# Supplementary material for: Slow integrin-dependent migration organizes networks of tissue-resident mast cells
Source: Nat Immunol. 2023 Apr 20;24(6):915–24. doi: 10.1038/s41590-023-01493-2 (PMC10232366; doi:10.1038/s41590-023-01493-2)
Supplement: Supplementary file 2 — Reporting Summary [file 41590_2023_1493_MOESM2_ESM.pdf]

nature portfolio

Corresponding author(s): Tim Lämmermann

Last updated by author(s): Mar 3, 2023

Reporting Summary

Nature Portfolio wishes to improve the reproducibility of the work that we publish. This form provides structure for consistency and transparency in reporting. For further information on Nature Portfolio policies, see our Editorial Policies and the Editorial Policy Checklist.

Statistics

For all statistical analyses, confirm that the following items are present in the figure legend, table legend, main text, or Methods section.

n/a	Confirmed	
☐	☒	The exact sample size (*n*) for each experimental group/condition, given as a discrete number and unit of measurement
☐	☒	A statement on whether measurements were taken from distinct samples or whether the same sample was measured repeatedly
☐	☒	The statistical test(s) used AND whether they are one- or two-sided<br>*Only common tests should be described solely by name; describe more complex techniques in the Methods section.*
☒	☐	A description of all covariates tested
☐	☒	A description of any assumptions or corrections, such as tests of normality and adjustment for multiple comparisons
☐	☒	A full description of the statistical parameters including central tendency (e.g. means) or other basic estimates (e.g. regression coefficient) AND variation (e.g. standard deviation) or associated estimates of uncertainty (e.g. confidence intervals)
☐	☒	For null hypothesis testing, the test statistic (e.g. *F*, *t*, *r*) with confidence intervals, effect sizes, degrees of freedom and *P* value noted<br>*Give P values as exact values whenever suitable.*
☒	☐	For Bayesian analysis, information on the choice of priors and Markov chain Monte Carlo settings
☒	☐	For hierarchical and complex designs, identification of the appropriate level for tests and full reporting of outcomes
☒	☐	Estimates of effect sizes (e.g. Cohen's *d*, Pearson's *r*), indicating how they were calculated

Our web collection on statistics for biologists contains articles on many of the points above.

Software and code

Policy information about availability of computer code

Data collection	For imaging data collection: Zen Blue (v 2012) and Zen Black (v8.1) (Carl Zeiss Microscopy)<br>For FACS data collection: FACSDiva (BD, v8.0.1).<br>For Western Blot data collection: Image Lab TM Touch Software (Bio-Rad, v6)
Data analysis	Kymographs were analyzed using a Matlab program which is available at GitHub (https://github.com/KMGlaser/Bambach-et-al.git). Mast cell clusters were analyzed using ClusterQuant2D. ClusterQuant2D code is provided at: https://zenodo.org/record/7684553.<br>Single-cell RNA sequencing analysis was done with the RaceID3 (v0.2.3) algorithm (Herman et al, 2018 Nat Methods 15, 379) and is available on CRAN (https://cran.r-project.org/) and RStudio (v4.0.2 GUI 1.72, source code as described previously, DOI: https://doi.org/10.1038/nature12175). Paired-end reads were aligned to the transcriptome using bwa (version 0.6.2-r126; https://bioweb.pasteur.fr/packages/pack@bwa@0.6.2).<br>For Imaging data analysis: Imaris (Bitplane, v9.1.2 and v9.5.1), Fiji (v2.1.0/1.53) and Huygens (SVI; v20.04)<br>For FACS data analysis: FlowJo (BD, v10.6.1)<br>For qPCR data analysis: Excel (Microsoft, v16)<br><br>For statistical data analysis and generation of graphs: Graphpad Prism (v9.3.1)

For manuscripts utilizing custom algorithms or software that are central to the research but not yet described in published literature, software must be made available to editors and reviewers. We strongly encourage code deposition in a community repository (e.g. GitHub). See the Nature Portfolio guidelines for submitting code & software for further information.

Data

Policy information about availability of data

All manuscripts must include a data availability statement. This statement should provide the following information, where applicable:

- Accession codes, unique identifiers, or web links for publicly available datasets
- A description of any restrictions on data availability
- For clinical datasets or third party data, please ensure that the statement adheres to our policy

Single-cell RNA-sequencing data have been deposited in Gene Expression Omnibus (GEO) with the accession code GSE205412 (https://www.ncbi.nlm.nih.gov/geo/query/acc.cgi?acc=GSE205412). Genome annotation ENCODE VM9 (https://hgdownload.soe.ucsc.edu/gbdb/mm10/) was used for mapping single-cell RNA-sequencing data. All other data supporting the findings of this study are included in the article, its supplementary materials or source data.

Human research participants

Policy information about studies involving human research participants and Sex and Gender in Research.

Reporting on sex and gender	This study doesn't include human research participants.
Population characteristics	N/A
Recruitment	N/A
Ethics oversight	N/A

Note that full information on the approval of the study protocol must also be provided in the manuscript.

Field-specific reporting

Please select the one below that is the best fit for your research. If you are not sure, read the appropriate sections before making your selection.

☒ Life sciences ☐ Behavioural & social sciences ☐ Ecological, evolutionary & environmental sciences

For a reference copy of the document with all sections, see nature.com/documents/nr-reporting-summary-flat.pdf

Life sciences study design

All studies must disclose on these points even when the disclosure is negative.

Sample size	No statistical methods were used to predetermine sample size. They were determined based on our previous publications, prior experience or pilot experiments.
Data exclusions	For most experiments: No data exlcusion methods were used. For image analysis of mast cell proximity to arterioles: Cells at ACTA2-positive structures without characteristic arteriolar morphology (capillaries) were manually excluded from the analysis.<br>Sc-RNAseq analysis: A very small cell subset showed additional macrophage features (cluster 10) (ED Fig. 5c), which we excluded from further analysis.
Replication	Reproducibility of the experimental findings was verified using biological replicates and independent experiments. The numbers are indicated in the respective figures (individual data points). Most experimental findings were replicated by independent experiments through several lab members.
Randomization	Experimental groups were defined by inhibitor treatment or by the genotype. Mast cells and mast cell clusters were randomly chosen for tracking, cell size analysis or cluster size determination, respectively.
Blinding	Experimentalists were blinded regarding the genotype of mice or BMMC cultures. Mouse numbers were used as identifiers.

Reporting for specific materials, systems and methods

We require information from authors about some types of materials, experimental systems and methods used in many studies. Here, indicate whether each material, system or method listed is relevant to your study. If you are not sure if a list item applies to your research, read the appropriate section before selecting a response.

Materials & experimental systems

n/a	Involved in the study
☐	☒ Antibodies
☐	☒ Eukaryotic cell lines
☒	☐ Palaeontology and archaeology
☐	☒ Animals and other organisms
☒	☐ Clinical data
☒	☐ Dual use research of concern

Methods

n/a	Involved in the study
☒	☐ ChIP-seq
☐	☒ Flow cytometry
☒	☐ MRI-based neuroimaging

Antibodies

Antibodies used

IgE coating of mast cells
Mouse Anti-Dinitrophenyl (DNP) IgE, clone: SPE-7; provided by Marc Schmidt-Supprian, TU Munich, Germany; used in all assays 1:300 (1ug/ml)

BMMC & PMC validation by FACS
Rat Anti-CD117 Brilliant Violet 421-conjugated; BioLegend; Cat# 105827; 1:200
Armenian Hamster Anti-FcεRIα Alexa Fluor 647-conjugated; BioLegend; Cat# 134309; 1:200

Fibronectin staining
Rabbit Anti-fibronectin; Sigma-Aldrich; Cat# F3648; 1:200
Donkey Anti-rabbit Alexa Fluor 488-conjugated; Invitrogen; Cat# A-21206; 1:400

Mast cell maturation and integrin expression
Rat Anti-CD16/CD32 Antibody; BD Biosciences; Cat# 553142; 1:250
Armenian Hamster Anti-FcεRIα Alexa Fluor 647-conjugated; BioLegend; Cat# 134309; 1:200
Armenian Hamster Anti-CD29 (Integrin beta 1) PE-conjugated; BioLegend; Cat# 102208; 1:400
Rat Anti-CD117 Brilliant Violet 421-conjugated; BioLegend; Cat# 105827; 1:800
Rat Anti-CD49f (Integrin alpha 6) Alexa Fluor® 488-conjugated; BioLegend; Cat# 313608; 1:400
Armenian Hamster IgG Isotype Control PE-conjugated; BioLegend; Cat# 400907; 1:400
Rat Anti-CD11b (Integrin alpha M) PE-conjugated; BD Biosciences; Cat# 557397; 1:400
Rat Anti-CD18 (Integrin beta 2) PE-conjugated; clone C71/16; BD Biosciences; Cat# 553293; 1:400
Rat Anti-CD49e (Integrin alpha 5); PE-conjugated, BD Biosciences; Cat# 557447; 1:400
Rat Anti-CD51 (Integrin alpha V) PE -conjugated BD; Biosciences; Cat# 551187; 1:400
Armenian Hamster Anti-CD61 (Integrin beta 3) PE-conjugated; BD Biosciences; Cat# 553347; 1:400
Rat Anti-Integrin β7 Chain PE-conjugated; BD Biosciences; Cat# 557498; 1:400
Rat Anti-CD41 (Integrin alpha IIb) FITC-conjugated; BD Bioscience; Cat# 553848; 1:400
Rat Anti-CD49d (Integrin alpha 4) PE-conjugated; Thermo Fisher Scientific; Cat# 12-0492-82; 1:400
Rat IgG2a kappa Isotype Control PE-conjugated; Thermo Fisher Scientific; Cat# 12-4321-80; 1:400
Rat IgG2b kappa Isotype Control PE-conjugated; Thermo Fisher Scientific; Cat# 12-4031-82; 1:400
Rat IgG2a Isotype Control Alexa Fluor 488-conjugated; Thermo Fisher Scientific; Cat# R2a20; 1:400
Rat IgG1 kappa Isotype Control FITC-conjugated; Thermo Fisher Scientific; Cat# 11-4301-81; 1:400
Rat Anti-CD18 (Integrin beta 2) FITC-conjugated; clone C71/16; BD Biosciences; Cat. 553292; 1:200
Rat Anti-CD29 Alexa Fluor 488 conjugated (Integrin beta 1, HMbeta1-1); BioLegend; Cat# 102211; 1:200
Rat IgG2b kappa isotype control FITC conjugated; ThermoFisher Scientific; Cat# 11-4031-82; 1:400
Rat Anti-CD117 APC-conjugated; ThermoFisher Scientific; Cat# 17-1172-83; 1:200
Armenian Hamster Anti-FcεRIα PE/CY7-conjugated; BioLegend; Cat# 134318; 1:200
Armenien Hamster IgG isotype control Alexa Fluor 488-conjugated; BD Bioscience; Cat# 53-4888-80; 1:200

Talin-1 depletion analysis
Mouse Anti-talin, clone:8d4; Sigma-Aldrich; Cat# T3287; 1:300 for FACS and 1:1000 for Western Blot
Donkey Anti-mouse Cy3TM-conjugated; Jackson ImmunoResearch Labs; Cat# 715-165-150; 1:800
Rabbit Anti-actin; Sigma-Aldrich; Cat# A2066; 1:2000
Rabbit Anti-mouse HRP-conjugated Antibody; Agilent Dako; Cat# P0161; 1:5000
Swine Anti-rabbit HRP-conjugated Antibody; Agilent Dako; Cat# P0217; 1:5000

Ear skin whole mount immunofluorescence analysis
Mouse Anti-smooth muscle actin (ACTA2) Alexa Fluor 405-conjugated; Abcam; Cat# ab210128; 1:500
Mouse Anti-smooth muscle actin (ACTA2) Cy3TM-conjugated; Sigma-Aldrich; Cat# C6198; 1:500
Rat anti-CD31; BD Bioscience; Cat# 550274; 1:150
Rabbit Anti-collagen IV; Abcam; Cat# ab19808; 1:500
Rat Anti-ACKR1 Alexa Fluor 488-conjugated, clone 6B7, provided by Aude Thiriot, Department of Immunology, Harvard Medical School, Boston; 1:300
Rabbit Anti-Desmin; Abcam; Cat# ab32362; 1:300
Rat Anti-endomucin; Invitrogen; Cat# 14-5851-81; 1:500
Rabbit Anti-fibronectin; Sigma-Aldrich; Cat# F3648; 1:200
Rabbit Anti-Lamc1; Sigma-Aldrich; Cat# HPA001909; 1:300
Goat Anti-Mcpt6; R&D Systems; Cat# AF3736; 1:600
Anti-MITF polyclonal; Atlas Antibodies; Cat# HPA003259; 1:100
Rabbit Anti-NG2; Sigma-Aldrich; Cat# AB5320; 1:200

Rat Anti-PDGFRa; Invitrogen; Cat# 14-4321-82; 1:200
Rabbit Anti-Vimentin; Abcam; Cat# ab92547; 1:300
Goat Anti-rabbit Alexa Fluor 405-conjugated; Thermo Fisher Scientific; Cat# A-31556; 1:300
Donkey Anti-goat Alexa Fluor 488-conjugated; Thermo Fisher Scientific; Cat# A-11055; 1:500
Donkey Anti-rabbit Alexa Fluor 568-conjugated; Thermo Fisher Scientific; Cat# A10042; 1:500
Donkey Anti-rabbit Alexa Fluor 488-conjugated; Invitrogen; Cat# A-21206; 1:500
Donkey Anti-rat Alexa Fluor 647-conjugated; Abcam; Cat# ab150155; 1:500
Donkey Anti-rat Alexa Fluor 405-conjugated; Abcam; Cat# ab175670; 1:500
Rabbit Anti-Red Fluorescent Protein (RFP); Rockland; Cat# 600-401-379; 1:1000
Goat Anti-GFP Dylight™ 488-conjugated; Rockland; Cat# 600-141-215; 1:1000

Immunofluorescence analysis of adhesion structures in mast cells
Mouse Anti-talin, clone:8d4; Sigma-Aldrich; Cat# T3287; 1:200
Mouse Anti-vinculin; Sigma-Aldrich; Cat# V9264; 1:200
Rat Anti-CD29 (Integrin beta 1, 9EG7); BD Biosciences; Cat# 553715; 1:200
Mouse Anti-paxillin; BD Biosciences; Cat# 610051; 1:200
Rat Anti-CD49e (Integrin alpha 5); BD Biosciences; Cat# 557446; 1:200
Goat Anti-rat Alexa Fluor 568-conjugated; Invitrogen; Cat# A-11077; 1:500
Goat Anti-mouse Alexa Fluor 647-conjugated; Invitrogen; Cat# A-21237; 1:500
Donkey Anti-mouse Cy3TM-conjugated; Jackson ImmunoResearch Labs; Cat# 715-165-150; 1:1000
Rabbit Anti-fibronectin; Sigma-Aldrich; Cat# F3648; 1:200
Goat Anti-rabbit Alexa Fluor 405-conjugated; Thermo Fisher Scientific; Cat# A-31556; 1:500
anti-rat CyTM3; Jackson ImmunoResearch; Cat: 712-166-153; 1:1000

RNA-FISH
Rat Anti-nidogen (NG2); Thermo Fisher; Cat# MA1-06501; 1:300
Donkey Anti-rat Alexa Fluor 405-conjugated; Abcam; Cat# ab175670; 1:500

FACS sort for single-cell RNA sequencing
Mouse Anti-CD45.2 Brilliant Violet 711™-conjugated; BioLegend; Cat# 109847; 1:100
Armenian Hamster Anti-CD3e PE-conjugated; Thermo Fisher Scientific; Cat# 2-0031-83; 1:100
Rat Anti-CD4; PE-conjugated BioLegend; Cat# 100407; 1:100
Rat Anti-CD8a PE-conjugated; Thermo Fisher Scientific; Cat# 12-0081-82; 1:100
Armenian Hamster Anti-CD11c (Integrin alpha X)PE-conjugated; BioLegend; Cat# 117307; 1:100
Rat Anti-CD19 PE-conjugated; Thermo Fisher Scientific; Cat# 12-0193-83; 1:100
Mouse Anti-NK-1.1 PE-conjugated; BioLegend; Cat# 108707; 1:100
Rat Anti-F4/80 PE-conjugated; Thermo Fisher Scientific; Cat# MF48004; 1:100

Validation

All commercial antibodies, validation informations can be found on the antibody registry (https://antibodyregistry.org) or company websites:
Armenian Hamster Anti-CD11c (Integrin alpha X)PE-conjugated: PRID: AB_313776
Armenian Hamster Anti-CD29 (Integrin beta 1) PE-conjugated: PRID: AB_312885
Armenian Hamster Anti-CD3e PE-conjugated: PRID:AB_465496
Armenian Hamster Anti-CD61 (Integrin beta 3) PE-conjugated: PRID: AB_394800
Armenian Hamster Anti-FcεRIα Alexa Fluor 647-conjugated: PRID: AB_1626097
Armenian Hamster Anti-FcεRIα PE/CY7-conjugated; PRID AB_10640122
Armenian Hamster IgG Isotype Control PE-conjugated: PRID: AB_326593
Donkey Anti-goat Alexa Fluor 488-conjugated: PRID: AB_2534102
Rabbit Anti-Red Fluorescent Protein (RFP): PRID:AB_2209751
Donkey Anti-mouse CyTM3-conjugated: PRID: AB_2340813
Donkey Anti-rabbit Alexa Fluor 488-conjugated: PRID: AB_2535792
Donkey Anti-rabbit Alexa Fluor 568-conjugated: PRID: AB_2534017
Donkey Anti-rat Alexa Fluor 405-conjugated: https://www.abcam.com/donkey-rat-igg-hl-alexa-fluor-405-preadsorbed-ab175670.html
Donkey Anti-rat Alexa Fluor 647-conjugated: PRID: AB_2813835
Goat Anti-GFP Dylight™ 488-conjugated: RRID: AB_1961516
Goat Anti-Mcpt6: PRID: AB_884330
Goat Anti-mouse Alexa Fluor 647-conjugated: PRID: AB_2535806
Goat Anti-rabbit Alexa Fluor 405-conjugated: PRID: AB_221605
Goat Anti-rat Alexa Fluor 568-conjugated: PRID: AB_2534121
Mouse Anti-CD45.2 Brilliant Violet 711™-conjugated: PRID: AB_2616859
Mouse Anti-Dinitrophenyl (DNP) IgE: Eshhar, Z., Ofarim, M. & Waks, T. Generation of hybridomas secreting murine reaginic antibodies of anti-DNP specificity. J Immunol 124, 775-780 (1980); PRID: AB_259249
Mouse Anti-NK-1.1 PE-conjugated; PRID: AB_313394
Mouse Anti-paxillin; PRID: AB_397463
Mouse Anti-smooth muscle actin Alexa Fluor 405-conjugated; https://www.abcam.com/alexa-fluor-405-alpha-smooth-muscle-actin-acetyl-e3--actg2-acetyl-e3-antibody-e184-ab210128.html
Mouse Anti-smooth muscle actin CyTM3-conjugated; PRID: AB_476856
Mouse Anti-vinculin; PRID: AB_10603627
Rabbit Anti-actin ; PRID: AB_476693
Rabbit Anti-collagen IV; RRID: AB_445160
Rabbit Anti-Desmin; PRID: AB_731901
Mouse Anti-talin; PRID: AB_477572

Rabbit Anti-fibronectin; PRID:AB_476976
Rabbit Anti-Lamc1; PRID: AB_1079230
Rabbit Anti-mouse HRP-conjugated Antibody; PRID: AB_2687969
Rabbit Anti-NG2; AB_11213678
Rabbit Anti-Vimentin; PRID: AB_10562134
Rat Anti-CD117 Brilliant Violet 421-conjugated; PRID: AB_10898120
Rat Anti-CD117 APC-conjugated; PRID AB_469434
Rat Anti-CD11b (Integrin alpha M) PE-conjugated; PRID: AB_396680
Rat Anti-CD16/CD32 Antibody; PRID:AB_394657
Rat Anti-CD18 (Integrin beta 2) PE-conjugated; clone C71/16, PRID: AB_394762
Rat Anti-CD18 (Integrin beta 2) FITC-conjugated; clone C71/16; PRID AB_394761
Rat Anti-CD19 PE-conjugated; PRID: AB_657660
Rat Anti-CD29 (Integrin beta 1, 9EG7); RRID:AB_395001
Rat Anti-CD29 Alexa Fluor 488 conjugated (Integrin beta 1, HMbeta1-1); PRID: AB_492830
Rat Anti-CD29 PE conjugated (Integrin beta 1, HMbeta1-1); PRID:AB_312884
Rat anti-CD31; PRID:AB_393571
Rat Anti-PDGFRa; RRID:AB_467491
Rat Anti-CD4 PE-conjugated; PRID: AB_312692
Rat Anti-CD41 (Integrin alpha IIb) FITC-conjugated; PRID: AB_395085
Rat Anti-CD49d (Integrin alpha 4) PE-conjugated; PRID: AB_465697
Rat Anti-CD49e (Integrin alpha 5); PRID: AB_396709
Rat Anti-CD49e (Integrin alpha 5) PE-conjugated; PRID: AB_396710
Rat Anti-CD49f (Integrin alpha 6) Alexa Fluor® 488-conjugated; PRID: AB_493635
Rat Anti-CD51 (Integrin alpha V) PE -conjugated; PRID: AB_394088

Rat Anti-CD8a PE-conjugated; PRID: AB_465530
Rat Anti-DARC Alexa Fluor 488-conjugated; Aude Thiriot, Carolina Perdomo, Guiying Cheng, Igor Novitzky-Basso, Sara McArdle, Jamie K. Kishimoto, Olga Barreiro, Irina Mazo, Robinson Triboulet, Klaus Ley, Antal Rot & Ulrich H. von Andrian. Differential DARC/ACKR1 expression distinguishes venular from non-venular endothelial cells in murine tissues. BMC Biol. 2017 May 19;15(1):45.
doi: 10.1186/s12915-017-0381-7.
Rat Anti-endomucin; PRID: AB_891529
Rat Anti-F4/80 PE-conjugated; PRID: AB_10372666
Rat Anti-Integrin β7 Chain PE-conjugated; PRID: AB_396735
Rat Anti-nidogen; PRID: AB_558792
Rat IgG1 kappa Isotype Control FITC-conjugated; PRID: AB_470008
Rat IgG1 kappa Isotype Control PE-conjugated; PRID: AB_470046
Rat IgG2a Isotype Control Alexa Fluor 488-conjugated; PRID: AB_2556535
Rat IgG2a kappa Isotype Control PE-conjugated; PRID: AB_1834380
Rat IgG2b kappa Isotype Control PE-conjugated; PRID: AB_470042
Swine Anti-rabbit HRP-conjugated Antibody; PRID: AB_2728719
Anti-MITF polyclonal; PRID: AB_1079381

Eukaryotic cell lines

Policy information about cell lines and Sex and Gender in Research

Cell line source(s)
CHO (Chinese hamster ovary) cells for SCF production; provided by Georg Häcker (University of Freiburg, Germany)
Immortalized MEFs (mouse embryonic fibroblasts); provided by Susanna Minguet (University of Freiburg, Germany)
WEHI-3 cells; provided by Rudolf Grosschedl (MPI of Immunobiology and Epigenetics, Freiburg, Germany); CVCL_3622

Authentication
The used previously reported immortalized cell lines were not further authenticated.

Mycoplasma contamination
The used cell lines were negative for Mycoplasma contamination, proven by PCR analysis.

Commonly misidentified lines (See ICLAC register)
No misidentified cell lines were used in this study.

Animals and other research organisms

Policy information about studies involving animals; ARRIVE guidelines recommended for reporting animal research, and Sex and Gender in Research

Laboratory animals
Mice between 7 to 12 weeks on C57BL/6J were used for this study. The following mouse strains were used in this study, detailed references are also given in the methods section of this manuscript:
Itgb1tm1Ref, MGI: 1926498;
Itgb2tm2Bay, MGI: 1861705;
Itgb3tm1.1Wlbcr, MGI: 5688572;
Commd10Tg(Vav1-iCre)A2Kio, MGI: 2449949
Gt(ROSA)26Sortm1(EYFP)Cos, MGI: 2449041 (also referred to as Rosa26-LSL:YFP);
Gt(ROSA)26Sor tm14(CAG-tdTomato)Hze, MGI: 3809524 (also referred to as Rosa26-LSL:Tom);
Tg(Myh11-cre,-EGFP)2Mik, MGI: 2653286;
Tyrc-2J (B6.Albino); MGI: 1855985
Tg(UBC-Brainbow1.0L)35Mal, MGI: 5645781

Kitltm1.1Sjm, MGI: 5300819
Tg(Kitl-ERT2Cre,-TdT), provided by Claus Nerlov (University of Oxford, United Kingdom), Buono et al 2016
Tg(CAG-EGFP)#Rows, MGI: 4831036
Tg(Cma1-cre)ARoer (brief: Mcpt5-Cre), MGI: 3785000
Tln1tm4.1Crit, MGI:3770513
Rag2tm1Fwa, MGI: 1858556
Mcpt5-Cre Tln1-fl/fl mice and crosses with fluorescent reporter lines (Rosa26LSL:YFP, Tg(Ubow)) were on a Tyrc-2J/c-2J (C57BL/6J-Albino) background, as we initially planned intravital microscopy studies of ear skin in these mice.

Wild animals	This study did not involve wild animals.
Reporting on sex	Both male and female mice were used for experiments. In comparative experiments, control and knockout mice were sex- and age-matched littermates.
Field-collected samples	No field-collected samples were used in this study.
Ethics oversight	Mouse breeding and husbandry were performed at the Max Planck Institute of Immunobiology and Epigenetics, Freiburg, in accordance with the guidelines provided by the Federation of European Laboratory Animal Science Association and as approved by German authorities (Regional Council of Freiburg). Mice were only used for organ removal after euthanasia by carbon dioxide exposure and thus not subject to experimental procedures and ethical approval according to §4 (3) Tierschutzgesetz.

Note that full information on the approval of the study protocol must also be provided in the manuscript.

Flow Cytometry

Plots

Confirm that:

☒ The axis labels state the marker and fluorochrome used (e.g. CD4-FITC).

☒ The axis scales are clearly visible. Include numbers along axes only for bottom left plot of group (a 'group' is an analysis of identical markers).

☒ All plots are contour plots with outliers or pseudocolor plots.

☒ A numerical value for number of cells or percentage (with statistics) is provided.

Methodology

Sample preparation	Details on sample preparation for flow cytometry are provided in the Methods section (Flow Cytometry)
Instrument	BD LSR Fortessa & BD LSRII
Software	Data collection: BD FACSDiva software<br>Data analysis: FlowJo software v10 (Tree Star, Ashland, OR, USA)
Cell population abundance	Sorted mast cells from skin were analyzed by single cell RNA sequencing and confirmed the expression of the mast cell marker Cpa3 of all sorted cells.
Gating strategy	Gating strategies have been included in Extended Data Figure 1 and 2.

☒ Tick this box to confirm that a figure exemplifying the gating strategy is provided in the Supplementary Information.
