# Supplementary material for: Slow integrin-dependent migration organizes networks of tissue-resident mast cells
Source: Nat Immunol. 2023 Apr 20;24(6):915–24. doi: 10.1038/s41590-023-01493-2 (PMC10232366; doi:10.1038/s41590-023-01493-2)
Supplement: Source Data Extended Data Fig. 2 — Unprocessed western blots. [file 41590_2023_1493_MOESM16_ESM.pdf]

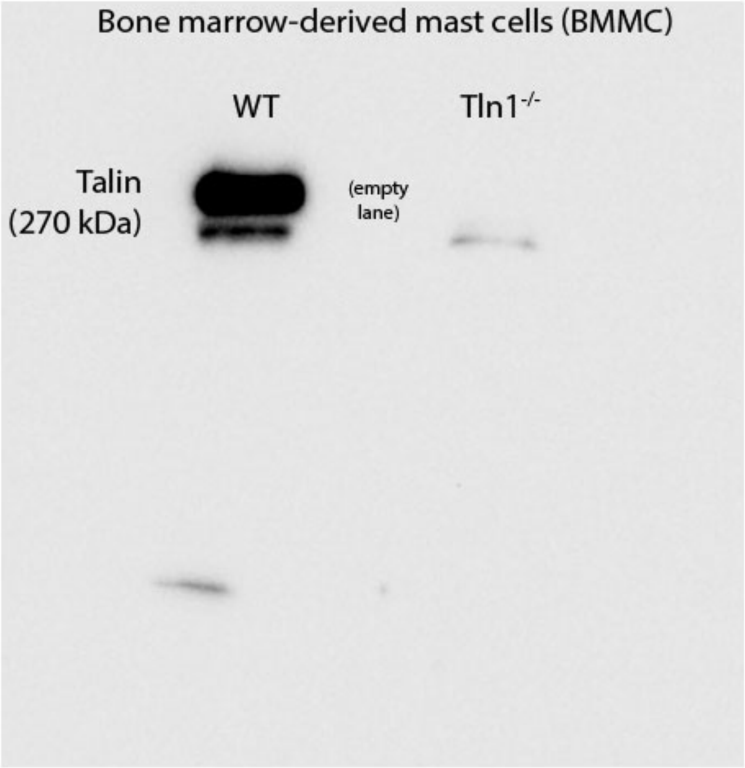
Bone marrow-derived mast cells (BMMC)
WT
Tln1-/-
Talin
(270 kDa)
(empty
lane)

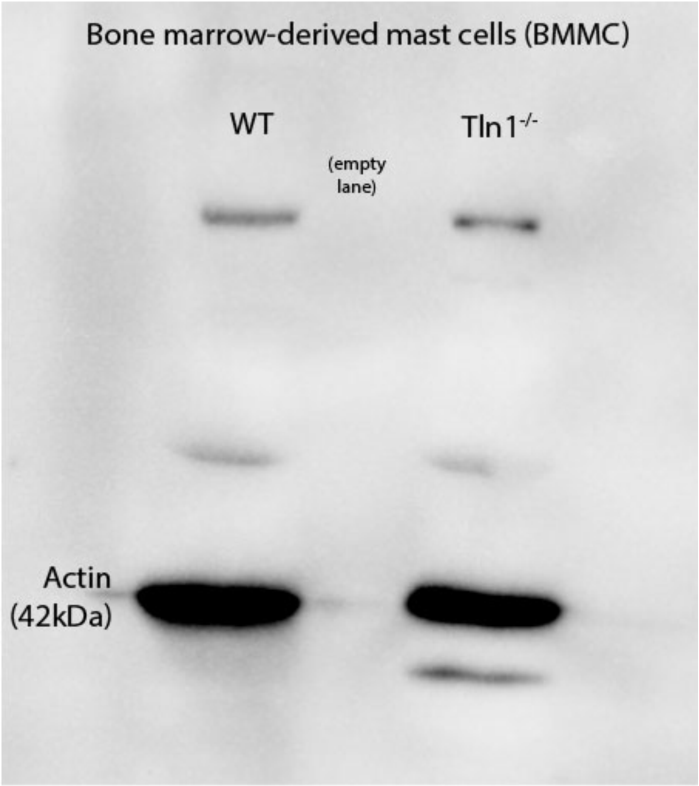
Bone marrow-derived mast cells (BMMC)
WT
Tln1-/-
(empty
lane)
Actin
(42kDa)

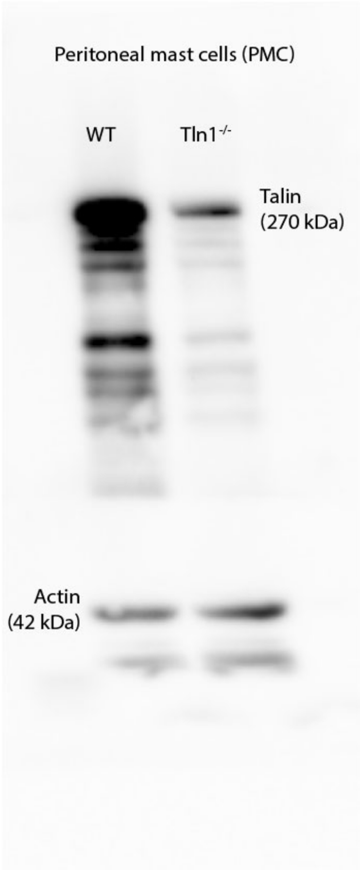
Peritoneal mast cells (PMC)
WT
Tln1-/-
Talin
(270 kDa)
Actin
(42 kDa)
